# Molecular typing of *Legionella pneumophila* isolates from environmental water samples and clinical samples using a five-gene sequence typing and standard Sequence-Based Typing

**DOI:** 10.1371/journal.pone.0190986

**Published:** 2018-02-01

**Authors:** Xiao-Yong Zhan, Qing-Yi Zhu

**Affiliations:** 1 Guangzhou KingMed Center for Clinical Laboratory, Guangzhou, China; 2 KingMed School of Laboratory Medicine, Guangzhou Medical University, Guangzhou, China; 3 The First Affiliated Hospital, Sun Yat-Sen University, Guangzhou, China; ENEA, Italian Agency for New Technologies, Energy and Sustainable Economic Development, ITALY

## Abstract

Inadequate discriminatory power to distinguish between *L*. *pneumophila* isolates, especially those belonging to disease-related prevalent sequence types (STs) such as ST1, ST36 and ST47, is an issue of SBT scheme. In this study, we developed a multilocus sequence typing (MLST) scheme based on two non-virulence loci (*trpA*, *cca*) and three virulence loci (*icmK*, *lspE*, *lssD*), to genotype 110 *L*. *pneumophila* isolates from various natural and artificial water sources in Guangdong province of China, and compared with the SBT. The isolates were assigned to 33 STs of the SBT and 91 new sequence types (nSTs) of the MLST. The indices of discrimination (IODs) of SBT and MLST were 0.920 and 0.985, respectively. Maximum likelihood trees of the concatenated SBT and MLST sequences both showed distinct phylogenetic relationships between the isolates from the two environments. More intragenic recombinations were detected in nSTs than in STs, and they were both more abundant in natural water isolates. We found out the MLST had a high discriminatory ability for the disease-associated ST1 isolates: 22 ST1 isolates were assigned to 19 nSTs. Furthermore, we assayed the discrimination of the MLST for 29 reference strains (19 clinical and 10 environmental). The clinical strains were assigned to eight STs and ten nSTs. The MLST could also subtype the prevalent clinical ST36 or ST47 strains: eight ST36 strains were subtyped into three nSTs and two ST47 strains were subtyped into two nSTs. We found different distribution patterns of nSTs between the environmental and clinical ST36 isolates, and between the outbreak clinical ST36 isolates and the sporadic clinical ST36 isolates. These results together revealed the MLST scheme could be used as part of a typing scheme that increased discrimination when necessary.

## Introduction

*Legionella pneumophila* (*L*. *pneumophila*) is a gram-negative bacterium worldwide in rivers and lakes as well as in many artificial water systems [1]. It is the major causative agent of Legionnaires’ disease (LD), which manifests as atypical pneumonia, Pontiac fever or a self-limited flu-like illness [2, 3]. Several molecular typing schemes have been used to investigate *L*. *pneumophila* epidemiology. These schemes included amplified fragment length polymorphism (AFLP), restriction fragment length polymorphism (RFLP), pulsed-field gel electrophoresis (PFGE), random amplified polymorphic DNA (RAPD) and Sequence-Based Typing (SBT). They have been used as part of a combinatory approach by some laboratories today [4–8]. The SBT, a scheme analogous to multilocus sequence typing (MLST), was proposed by the European Working Group for *Legionella* Infections (EWGLI, now is the ESCMID Study Group for *Legionella* Infections, ESGLI). It is an essentially seven-locus sequence typing method performed by sequencing and comparing seven loci (*flaA*, *pilE*, *asd*, *mip*, *mompS*, *proA*, and *neuA*), and appears to be a powerful tool for global epidemiology [9, 10]. MLST approach with nonselective housekeeping genes has been well documented [11], while a combination of selective targets could produce sufficient discrimination to allow epidemiological typing of *L*. *pneumophila* [9]. Gaia has first chosen seven genes, including four non-selective (*acn*, *groES*, *groEL*, and *recA*) and three selective (*flaA*, *proA*, and *mompS*) to determine the availability of these genes in investigating the outbreaks of LD caused by *L*. *pneumophila* [12]. Then a modified six-gene (*flaA*, *proA*, *mompS*, *asd*, *mip*, and *pilE*) sequence typing was performed to improve the previous method [9]. In 2007, *neuA* was added to the six-gene sequence typing. It increased the discriminatory ability of the consensus sequence-based scheme for typing *L*. *pneumophila* and eventually formed the SBT scheme [10]. Although SBT is the current “gold standard” typing method for investigation of LD outbreaks, however, as common sequence types (STs) such as ST1, ST47, and ST36 isolates cause many infections, some investigations remain unresolved [13]. For example, subtyping the isolates belonging to a same prevalent ST required combinatory approach, including PFGE, AFLP, monoclonal antibody-based (MAb) subgrouping and some other genome sequence-based typing schemes [14–16]. A large proportion of LD cases is caused by just a small number of common STs (e.g., ST1); the SBT can lack discriminatory power [13, 17, 18]. Therefore, research and improvement of molecular typing methods for *L*. *pneumophila* are desirable.

As an opportunistic bacterium that inhabits aquatic environments, *L*. *pneumophila* has an intra-amoebal lifestyle. Free-living amoeba in natural water environments is the reservoir and shelter for *L*. *pneumophila*. From the natural water, it can colonize the artificial water environments such as cooling towers and hot-water systems and then spread in aerosols, infecting the susceptible person [19, 20]. So far, person-to-person transmission of *L*. *pneumophila* has rarely been reported, the infection of LD is mainly via the inhalation of *Legionella*-containing aerosols [21, 22]. Thus aquatic environments could serve as potential sources of *Legionella* infection, and epidemiological study of environmental isolates was of great importance. In a previous study, we researched the genetic diversity of clinical, artificial and natural water isolates at the non-virulence gene and virulence gene levels, respectively [23]. Five gene loci including two non-virulence loci (tryptophan synthase α subunit-encoding gene, *trpA* and tRNA nucleotidyltransferase gene, *cca*), which are common in a set of bacterial genomes, and three virulence loci (*icmK*, *lspE*, and *lssD*) belonging to the components of different secretion systems were studied. The allelic diversities of these loci in our environmental isolates implied that an MLST scheme based on these loci seemed to yield high discriminatory ability for these isolates. Therefore, we developed a five-gene (*cca*, *trpA*, *lspE*, *lssD*, and *icmK*) MLST scheme. The aims of this study were;

1. To evaluate the discriminatory power of the MLST scheme in genotyping 110 *L*. *pneumophila* isolates from various natural and artificial water sources of Guangdong Province of China, and compared it with the SBT scheme. This would answer whether the MLST could provide a higher discrimination for environmental isolates.
2. To investigate the diversity of the *L*. *pneumophila* isolates from natural and artificial water sources based on ST and new sequence type (nST, sequence type of the MLST) distributions. The phylogeny and molecular evolution of these isolates based on SBT sequences, and MLST sequences were also investigated to probe possible mechanism that operated the ST and nST distributions in different water sources. These would enable comparison of the genetic types in these isolates determined by SBT with that derived by MLST and enable the analysis of correspondence between the MLST and SBT schemes.
3. To determine the potential of the MLST scheme in genotyping reference clinical and environmental *L*. *pneumophila* strains, especially those strains with prevalent STs. We would try to find whether there were different distribution patterns of nSTs between the environmental and clinical isolates, and between the outbreak and sporadic clinical isolates.

## Materials and methods

### Ethics statement

The local Centers for Disease Control and Prevention (CDC) and the hotel managers authorized the collection of cooling tower water of the hotels. There were no specific permissions required for the collection of water samples from lakes, rivers, and ponds; because they were public open areas for citizens. Our study did not involve endangered or protected species.

### *L*. *pneumophila* isolates

Our environmental collection included 51 artificial water isolates and 59 natural water isolates. They were isolated from ponds, rivers, lakes and air conditioning cooling towers in 14 different sites in Guangdong Province of China, between October 2003 and September 2007. The details of the isolates including the locations where they were isolated, the geographic coordinates, and the collection dates, were summarized in S1 Table. These isolates were used to investigate the discriminatory ability of the MLST scheme for the environmental *L*. *pneumophila* isolates, and to investigate the diversity, the phylogeny and molecular evolution of the isolates from natural and artificial water sources. All identified *Legionella* isolates were grown on buffered charcoal yeast extract (BCYE) agar plates at 37°C with 5% CO_2_ for three days, and then the bacteria cultures were harvested. Genomic DNA extraction was performed as shown in our previous report [24].

Besides our environmental isolates, we used 19 reference clinical strains belonging to prevalent STs to investigate the genotyping potential of the MLST scheme. Ten reference environmental strains belonging to a prevalent ST (ST36) were also used to assess the discriminatory ability of the MLST for the isolates with the same ST but from different sources (clinical and environmental sources). The details of these strains are shown in Table 1.

**Table 1:** *L*. *pneumophila* reference clinical and environmental strain information.

Strain*	Source nature	Geographic location	Collection year	NCBI BioSample No.	GenBank accession No.	ST^a^	nST
Thunder Bay	Clinical	Canada	N/A	SAMN02603729	CP003730.1	187	92
ATCC43290	Clinical	USA: Denver	1987	SAMN02603182	NC_016811.1	187	92
ERS1434278	Clinical	Germany	1999	SAMEA4535099	NZ_LT632617.1	42	93
Lorraine	Clinical	France: Lorraine	N/A	SAMEA3138425	NC_018139.1	47	94
130b	Clinical	USA	1980s	SAMEA2272454	FR687201.1	42	93
LP_617	Clinical	UK	2003	SAMEA1487522	NZ_FJOC01000001.1- NZ_FJOC010000074.1#	47	100
Lens^b^	Clinical	France: Lens	2003	SAMEA3138253	NC_006369.1	15	95
Alcoy	Clinical	Spain: Alcoy	1999	SAMN02604292	NC_014125.1	578	96
Corby	Clinical	United Kingdom	N/A	SAMN02603241	NC_009494.2	51	97
OLDA	Clinical	USA	1947	SAMN05198688	CP016030.2	1	20
Paris	Clinical	France: Paris	1987	SAMEA3138252	NC_006368.1	1	20
C1_S	Clinical	USA: North Carolina	2009	SAMN05179547	CP015932.1	36	92
C2_S	Clinical	USA: Illinois	2007	SAMN05179997	CP015933.1	36	92
C3_O	Clinical	USA: Texas	2006	SAMN05180024	CP015934.1	36	92
C5_P	Clinical	USA: Ohio	1998	SAMN05180026	CP015936.1	36	92
C7_O	Clinical	USA: Deleware	1994	SAMN05180028	CP015938.1	36	98
C9_S	Clinical	USA: Indiana	1982	SAMN05180030	CP015941.1	36	92
C10_S	Clinical	USA: Nebraska	1990	SAMN05180031	CP015944.1	36	99
Philadelphia_1	Clinical	USA: Pennsylvania	1977	SAMN05180044	CP015928.1	36	92
E1_P	Environmental	USA: California	2013	SAMN05180033	CP015946.1	36	92
E2_N	Environmental	USA: Nevada	2012	SAMN05180034	CP015947.1	36	92
E3_N	Environmental	USA: Texas	2011	SAMN05180035	CP015949.1	36	92
E4_N	Environmental	USA: Alabama	2012	SAMN05180036	CP015950.1	36	92
E5_N	Environmental	USA: Arkansas	2011	SAMN05180037	CP015951.1	36	92
E6_N	Environmental	USA: New Jersey	2011	SAMN0518003	CP015953.1	36	101
E7_O	Environmental	USA: Georgia	2009	SAMN05180039	CP015954.1	36	92
E8_O	Environmental	USA: Texas	2006	SAMN05180040	CP015955.1	36	3
E9_O	Environmental	USA: Illinois	2012	SAMN05180041	CP015956.1	36	92
E10_P	Environmental	USA: Ohio	2007	SAMN05180042	CP015925.1	36	92

* The clinical and environmental ST36 isolates in the present study included the confirmed outbreak-associated isolates (_O), the potential outbreak isolates (_P), the sporadic isolates (_S), the non-disease-associated isolates (_N) and Philadelphia-1 isolates from USA CDC (Philadelphia_1). C1_S, C2_S, C3_O etc. indicate clinical isolates, while E1_P, E2_N, E3_N etc. indicate environmental isolates.

# Strain LP_617 only showed a set of whole genome shotgun sequences.

a Sequence type was derived from the genome sequence data.

b Strain Lens has two non-identical copies of the *mompS* locus (354nt) in their genome, and its ST was defined according to Moran-Gilad’s report [16].

### Five-gene MLST and SBT schemes

All the environmental isolates were selected for sequencing partial *cca*, *trpA*, *lspE*, *lssD* and *icmK* genes. We selected the most variable regions through a sequence alignment with the known sequences (including sequences from reference *L*. *pneumophila* strains, such as Thunder Bay, ATCC43290, Lens, Alcoy, Corby, etc.) in the NCBI database in order to achieve maximum genetic variability and to make it represents the allelic diversity of these genes. The genes, reference gene ID of the NCBI database, primers, the fragment sizes of the PCR products, the gene regions used for the analysis, and the number of alleles found during this study are shown in S2 Table. PCR was employed to amplify fragments of DNA. The PCR was performed using a 2×EasyPfu PCR SuperMix (Transgene Biotech, Beijing) with 0.1 U Pfu polymerase/μl, 500 μM dNTP each, 50 mM Tris-HCl (pH8.7), 20 mM KCl, and 4 mM MgCl in a ready-to-use formulation. Primers were added to a total volume of 25 μl with a final concentration of 200 nM. PCR was carried out using the GeneAmp PCR system (MJ Research PTC-200) with the following thermal conditions: 95 ^o^C for 3 min, followed by 35 cycles of 95 ^o^C for 20 s, 60 ^o^C for 20 s and 72 ^o^C for 30 s (*lspE*, *lssD*, and *icmK* loci) or 70 s (*cca*, and *trpA* loci), and a final extension at 72 ^o^C for 5 min. For confirmation, each PCR reaction was performed with a positive control (*L*. *pneumophila* strain ATCC33152 genomic DNA as the PCR template) and a negative control (sterile water as the PCR template). PCR products were purified by an EasyPure Quick Gel Extraction Kit (Transgene Biotech, Beijing) and then transferred to Guangzhou IGE Biotechnology Ltd for sequencing.

The quality of DNA sequencing was manually checked by Chromas (http://technelysium.com.au). The gene regions assembled to form a concatenated MLST sequence were shown in S2 Table. An nST was defined as a new allele of the concatenated MLST sequence. The STs were determined by using the protocol from ESGLI with seven gene fragments (*flaA*, *pilE*, *asd*, *mip*, *mompS*, *proA*, and *neuA*) according to the standard process shown in *L*. *pneumophila* SBT website (http://www.hpa-bioinformatics.org.uk/legionella/legionella_sbt/php/sbt_homepage.php). The sequences of the SBT loci and MLST loci of the 29 reference *L*. *pneumophila* strains were gained from NCBI database. Their nSTs and STs were determined by analyzing the concatenated MLST and SBT sequences (Table 1).

### Population genetic analysis

The indices of discrimination (IODs) of the SBT and MLST for the isolate collection were calculated using Simpson’s index of diversity, as first described by Hunter and Gaston [25]. DnaSP 5.10.01 was used to perform genetic diversity analyses of the concatenated MLST and SBT sequences of the environmental isolates [26, 27]. The proportion of each nST or ST was compared between the natural and artificial water isolates by using Fisher’s exact test or Chi-Square test (SPSS 16.0, SPSS Inc., USA). Analysis of molecular variance (AMOVA) for the concatenated MLST sequences and SBT sequences was performed with Arlequin Ver3.5.2 [28]. We defined the hierarchical subdivision of the environmental isolates at three levels. At the upper level, the two groups considered were based on the two cities where they were isolated (Guangzhou and Jiangmen groups, consisted of 66 and 44 isolates, respectively). As populations within groups, the intermediate level, we reckoned the isolates from the same environment as subpopulations. Therefore, Guangzhou and Jiangmen groups of isolates were both split into two subgroups (natural and artificial water subpopulations). The third level corresponded to the different haplotypes which were found within the four subgroups considered in the previous level.

### Phylogenetic analysis

Phylogenetic analysis was conducted by MEGA7 package [29]. Maximum likelihood (ML) trees were obtained for the concatenated MLST and SBT sequences separately with MEGA7, based on the Kimura 2-parameter model [30]. Initial tree(s) was obtained automatically by applying Neighbor-Join and BioNJ algorithms to a matrix of pairwise distances estimated using the Maximum Composite Likelihood (MCL) approach. ML tree nodes were evaluated by bootstrapping with 1000 replications.

### Molecular evolution analysis

The neighbor-net analysis was performed and converted to a splits graph using the drawing algorithms implemented in SplitsTree4 software (version 4.14.4) [31, 32]. A reticulate network tree was prepared to show the relationships among different STs or nSTs and to visualize possible recombination events.

The concatenated MLST and SBT sequences of our environmental isolates were screened using RDP4 to detect intragenic recombinations [33]. Six methods implemented in the program RDP4 were utilized. These methods were RDP [34], GENECONV, BootScan [35], MaxChi [36], Chimaera [37], and SiScan [38]. Potential recombination events (PREs) were considered as those identified by at least two methods according to Coscolla’s report [39]. Common settings for all methods were to consider sequences as linear, statistical significance was set at the P < 0.05 level, with Bonferroni correction for multiple comparisons and requiring phylogenetic evidence and polishing of breakpoints.

### Nucleotide sequence accession numbers

The 550 sequences of the five MLST loci from the 110 *L*. *pneumophila* environmental isolates determined in this study were deposited in the GenBank Nucleotide Sequence Database with Accession No. KY708328-KY708437 (*cca*), KY708438-KY708547 (*trpA*), KY708658- KY708767 (*lspE*), KY708768- KY708877 (*lssD*), and KY708548- KY708657 (*icmK*).

## Results and discussion

### *L*. *pneumophila* five-gene MLST and SBT for environmental isolates

Of the 110 isolates, 33 STs of the SBT and 91 nSTs (the 91 nSTs were designated to be nST1, nST2, nST3 etc.) of the MLST were assigned (Table 2 and Table 3). The most dominant ST was ST1, which accounted for 20% (22/110) of all *L*. *pneumophila* isolates, and mostly came from the artificial water sources (Table 2). ST1, the most prevalent and disease-associated ST worldwide, was also the most abundant in the EWGLI SBT database, followed by ST23 and ST47 [40]. ST1048, another dominant ST identified in this study, constituted 11.82% (13/110) of all isolates. Sixteen STs included only one isolate. The proportions of ST1 and ST1054 isolates were significantly higher in artificial environments (Fisher’s exact test, P < 0.001 and P = 0.043, respectively), while the proportions of ST1048, ST739, and ST1267 isolates were higher in natural environments (Fisher’s exact test, P = 0.006, P = 0.014, and P = 0.029, respectively). These findings reinforced the evidence that the distribution of STs between the natural and artificial environments was distinct [41].

**Table 2:** ST distributions in the isolates from natural and artificial water sources.

ST	Allelic profile	Natural isolates		Artificial isolates		*P-*value (Fisher’s exact test)
		*n*	*%*	*n*	*%*	
ST1	1, 4, 3, 1, 1, 1, 1	2	3.39	20	39.22	**<0.001**
ST630	1, 4, 3, 1, 1, 1, 10	4	6.78	2	3.92	0.684
ST1417	8, 6, 34, 9, 2, 8, 209	2	3.39	3	5.88	0.664
ST242	3, 10, 1, 28, 1, 9, 3	1	1.69	3	5.88	0.338
ST1048	6, 10, 17, 3, 4, 14, 11	12	20.34	1	1.96	**0.006**
ST59	7, 6, 17, 3, 13, 11, 11	0	0	2	3.92	0.213
ST739	12, 8, 11, 2, 10, 12, 2	7	11.86	0	0	**0.014**
ST1267	2, 6, 48, 6, 48, 5, 40	6	10.17	0	0	**0.029**
ST1266	12, 15, 11, 56, 29, 12, 34	2	3.39	0	0	0.496
ST1785	2, 15, 3, 73, 29, 1, 201	2	3.39	0	0	0.496
ST45	5, 1, 22, 26, 6, 10, 12, 45	2	3.39	0	0	0.496
ST1049	12, 8, 11, 2, 11, 12, 4	2	3.39	0	0	0.496
ST752	22, 4, 3, 1, 1, 1, 1	2	3.39	7	13.73	0.078
ST1052	2, 10, 15, 28, 21, 3, 2	2	3.39	0	0	0.496
ST1053	6, 16, 14, 28, 4, 14, 3	3	5.08	0	0	0.247
ST1777	1, 4, 3, 1, 1, 1, 215	2	3.39	1	1.963	1.000
ST1054	32, 12, 50, 6, 48, 11, 9	0	0	4	7.843	**0.043**
Other STs*		8	13.56	8	15.69	0.752(Chi-Square test)
Total		59	100	51	100	

* Other STs, 16 STs including only one isolate

**Table 3:** nST distributions in the isolates from natural and artificial water sources.

Five-gene MLST	Natural isolates		Artificial isolates		*P-*value (Fisher’s exact test)
	*n*	*%*	*n*	*%*	
nST5	0	0	2	3.92	0.213
nST15	0	0	3	5.88	0.096
nST17	0	0	2	3.92	0.213
nST20	1	1.69	2	3.92	0.596
nST35	2	2.39	2	3.92	1.000
nST39	0	0	5	9.80	**0.019**
nST50	5	8.47	0	0	0.060
nST68	2	2.39	0	0	0.498
nST82	2	2.39	0	0	0.498
Other nSTs*	47	79.66	35	68.63	0.185 (Chi-Square test)
Total	59	100	51	100	

* Other nSTs, 82 nSTs including only one isolate

NST50 and nST39 were the prevalent nSTs in this study (Table 3), but only constituted 4.55% (5/110) of all isolates. Most of the nSTs included only one isolate (90.11%, 82/91). The proportion of nST39 was significantly higher in artificial environments (Fisher’s exact test, P = 0.019). The allele diversity of the seven SBT loci (*flaA*, *pilE*, *asd*, *mip*, *mompS*, *proA*, and *neuA*) in these isolates ranged from 9 to 17, while the allele diversity of the five MLST loci ranged from 12 to 18 in *cca*, *trpA*, *lssD*, *lspE* locus and the significant 83 in *icmK* locus (S2 Table). The 91 nSTs in 110 isolates implied higher discriminatory power of the MLST than that 33 STs in 110 isolates (IOD = 0.985 vs. IOD = 0.920, S3 Table). David and colleague studied the diversity of 79 epidemiologically unrelated *L*. *pneumophia* isolates. The IODs of these isolates were 0.972, 0.991 and 0.940 through the using of a 53 ribosomal-gene MLST (rMLST), a 100 core-gene MLST (cgMLST), and the SBT, respectively [13]. The discriminatory power of the five-gene MLST scheme might be similar to the 100 core-gene cgMLST scheme [13].

### Diversity of the *L*. *pneumophila* isolates from natural and artificial water sources based on the MLST and the SBT schemes

Table 2 and Table 3 show the ST and nST compositions of the *L*. *pneumophila* isolates recovered from natural and artificial water sources. Fifty-nine isolates from natural water sources were grouped into 52 nSTs, and 51 artificial water isolates were grouped into 41 nSTs; while they were grouped into 23 STs and 17 STs, respectively. The diversity of nSTs was higher in the isolates from natural water sources than in those from artificial ones (IOD = 0.973 vs. IOD = 0.902, S3 Table). Similarly, the diversity of STs was also higher in the isolates from natural water sources (IOD = 0.914 vs. IOD = 0.807, S3 Table). Many studies demonstrated that diversity of isolates from natural water sources was higher than those from artificial water sources, but these studies were based on ST distributions [42, 43]. In the present study, we obtained similar results not only based on ST distributions but also based on nST distributions and the diversity of nSTs in these isolates was higher than that of STs, indicating the MLST scheme was efficiency in determining the diversity of *L*. *pneumophila* isolates from different water sources. Moreover, we analyzed the genetic diversity of these isolates based on the concatenated SBT and MLST sequences. It showed that genetic diversity parameters such as haplotypes, haplotype diversity, nucleotide diversity, and nucleotide differences, were higher in the isolates from natural water sources (Table 4). Most of these parameters derived from the MLST sequences were also higher. This result was in accord with our observation in the diversities of nSTs and STs and implied the five-gene MLST scheme had higher discriminatory ability than the SBT scheme.

**Table 4:** Genetic diversity of the concatenated MLST and SBT sequences in *L*. *pneumophila* isolates from natural (N) and artificial (A) water sources.

Sequences	Strain types	Sequence, n	Sequence length	*h*	*Hd*	SD of Hd	*π*	SD of *π*	*S*	*θ*	SD of *θ*	*k*	*ƞ*
MLST	N	59	2876	**52**	**0.992**	0.006	**0.02689**	0.00121	312	0.02335	0.00631	**77.328**	354
	A	51	2876	41	0.987	0.008	0.02507	0.00502	380	0.02937	0.00814	72.100	415
	All	110	2876	**91**	**0.994**	0.003	0.02763	0.00243	485	0.03198	0.00767	**79.469**	547
SBT	N	59	2501/2498	**23**	**0.930**	0.019	**0.03072**	0.00338	300	0.02585	0.00699	**76.731**	355
	A	51	2501	17	0.824	0.047	0.02217	0.00402	316	0.02808	0.00780	55.454	338
	All	110	2501/2498	33	0.928	0.013	0.02807	0.00252	369	0.02801	0.00675	70.118	429

*H*, Haplotypes,

*Hd*, Haplotype diversity

*π*, Nucleotide diversity

*S*, Polymorphic sites

*θ*, Theta (per site) from S

*k*, Nucleotide differences

*Ƞ*, Total number of mutations

Besides IOD comparison, we also performed a hierarchical AMOVA analysis to study the genetic variation of the concatenated MLST and SBT sequences in these isolates. The largest proportion of the genetic variation was found within populations, as this level accounted for 89.78% of the total variation in the MLST sequences, and 89.75% of the total variation in the SBT sequences (Table 5). The fixation indices among groups (F_CT_) were -0.0444 (MLST sequences) and -0.05784 (SBT sequences), and the variation did not vary significantly among the groups (P = 1.00), indicating no different genetic diversities of the isolates from the two cities exists. In contrast, fixation indices among populations (F_SC_) were 0.14038 (MLST sequences) and 0.10249 (SBT sequences), and the genetic variation varied significantly among populations within groups (P < 0.01, Table 5). These results supported the notion that genetic differentiation existed between the isolates from the natural and artificial water sources, and *L*. *pneumophila* isolates from natural water sources had more genetic diversities.

**Table 5:** Analysis of molecular variance of concatenated MLST and SBT sequences.

Sequences	Source of variation	d.f.	Sum of squares	Variance components	Percentage of variation	F-statistics
MLST	Among groups	1	170.327	-1.82081 Va	-4.44	*FCT = -0*.*04444*
	Among populations within groups	2	261.272	6.00777 Vb	14.66	*FSC = 0*.14038**
	Within populations	106	3899.455	36.78731 Vc	89.78	*FST = 0*.10219**
	Total	109	4331.055	40.97427		
SBT	Among groups	1	144.336	-2.10141 Va	-5.78	*FCT = -0*.*05784*
	Among populations within groups	2	247.195	5.82481 Vb	16.03	*FSC = 0*.10249**
	Within populations	106	3456.305	32.60666 Vc	89.75	*FST = 0*.15156**
	Total	109	3847.836	36.33005		

** P < 0.01

### Phylogeny of environmental *L*. *pneumophila* isolates based on concatenated MLST sequences of the 91 nSTs and SBT sequence of the 33 STs

**Fig 1:**
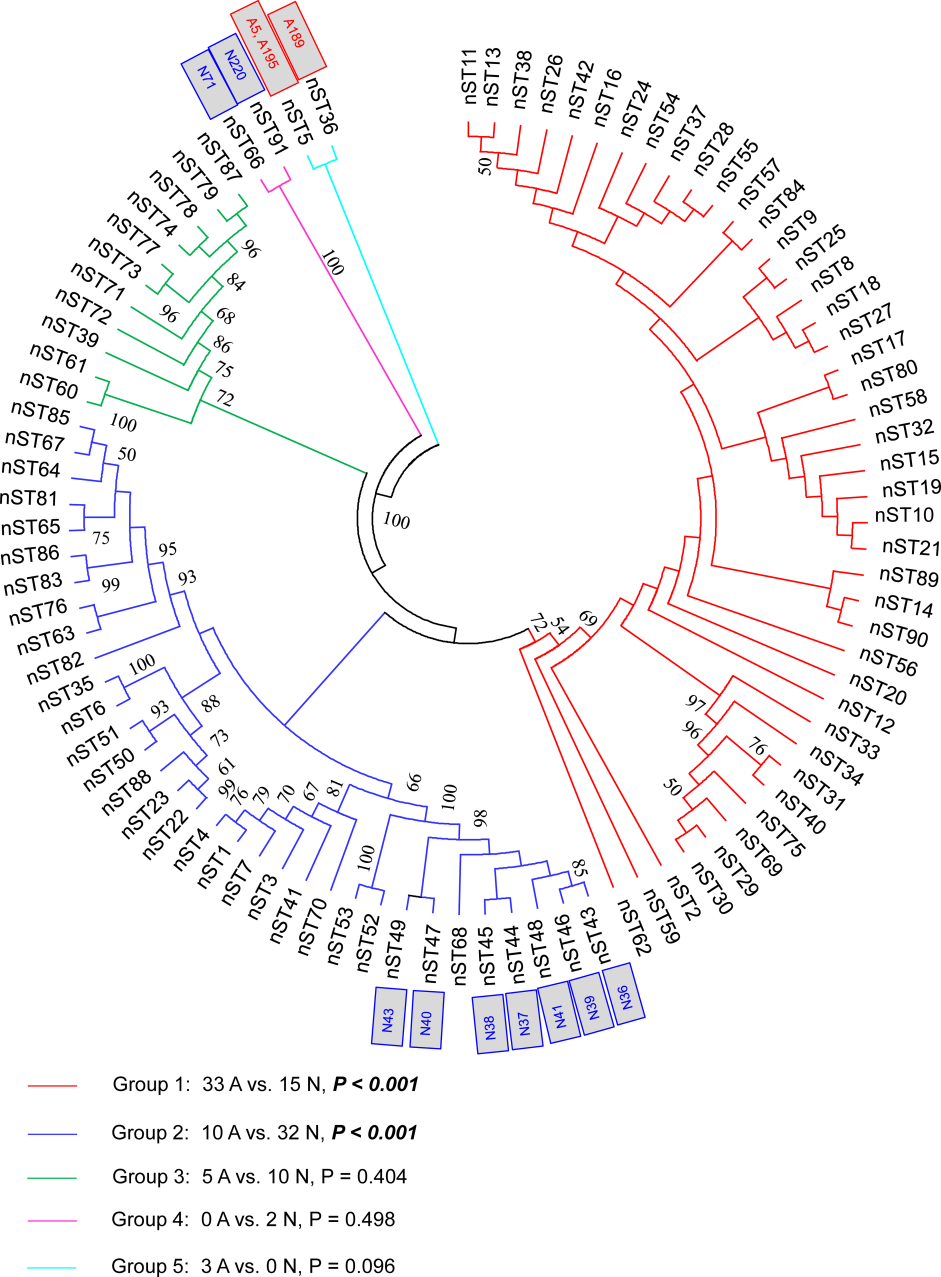
Phylogenetic tree of the concatenated MLST sequences (2876 bp) of the 91 nSTs in this study. Bootstrap support values (1000 replicates) for nodes higher than 50% are indicated next to the corresponding node. Five main groups of the branches could be found. Different color of the branches indicated distinct groups of the nSTs, and branches with the same color were clustered into a group. The blocks indicate the strains of corresponding nSTs. A indicates artificial isolates, and N indicates natural isolates.

**Fig 2:**
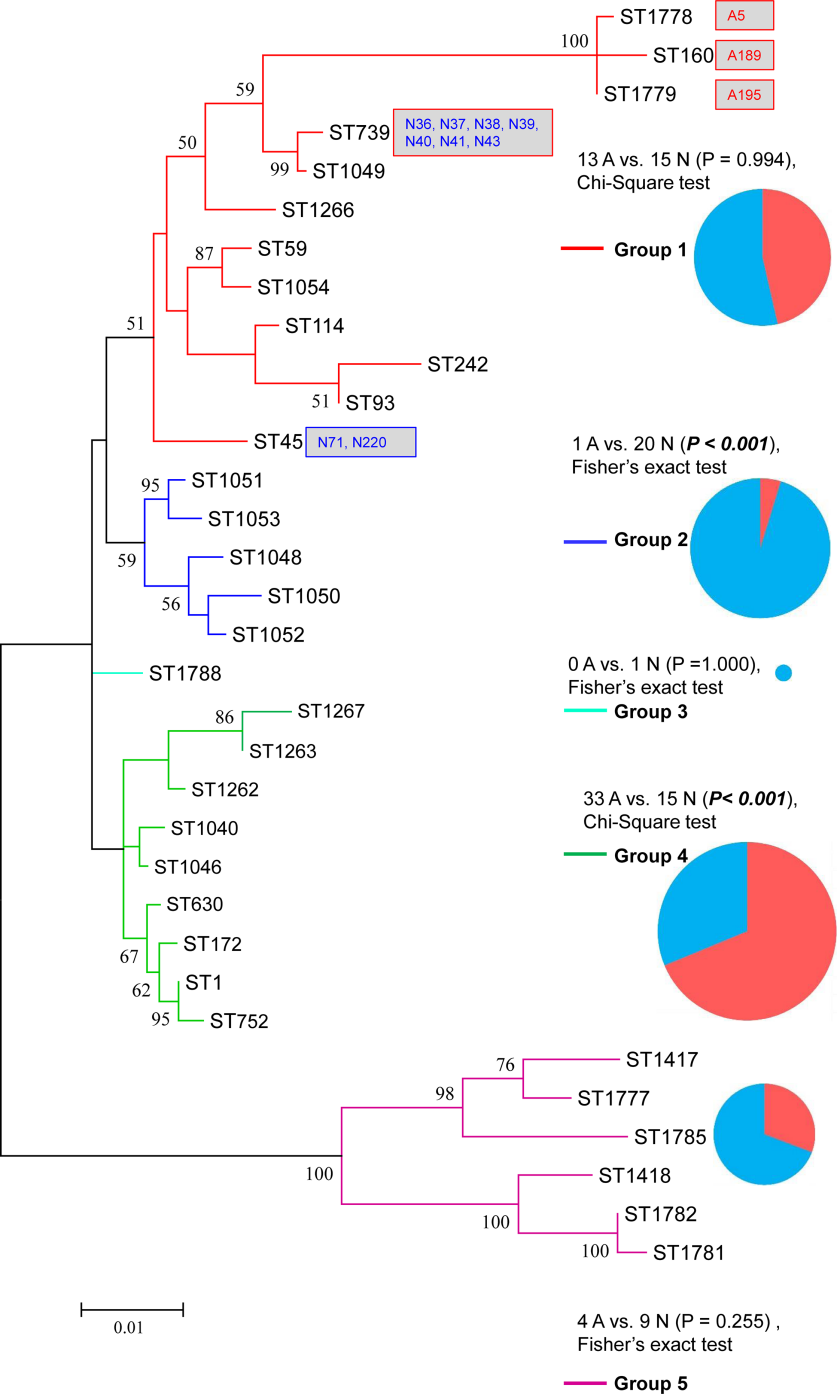
Phylogenetic tree of concatenated SBT sequences (2501/2498bp) of the 33 STs in this study. Bootstrap support values (1000 replicates) for nodes higher than 50% are indicated next to the corresponding node. Five main groups of the branches could be found. Different color of the branches indicates distinct groups of the nSTs. Branches with the same color are clustered into a group. The relative size of solid circles indicates the number of isolates in the selective group; the red sector indicates artificial water isolates, while the blue sector indicates natural water isolates. The blocks indicate the isolates of the corresponding STs. A indicates artificial water isolates, and N indicates natural water isolates.

ML tree of the concatenated MLST sequences of the 91 nSTs showed five main groups: forty-three nSTs formed nST group 1, and the isolates within this group were mainly from artificial water sources (68.75%, 33/48, P < 0.001, Chi-Square test); while 32 nSTs formed nST group 2, and the isolates within this group were mainly from natural water sources (76.19%, 32/42, P < 0.001, Chi-Square test) (S4 Table, Fig 1). We also found a comparable result in the ML tree of the concatenated SBT sequences of the 33 STs (Fig 2). ST1788, which only included one natural isolate (N67, an nST62 isolate), constituted a group. Of the five STs in group 2, the isolates of this group were mainly from natural water sources (95.24%, 20/21, P < 0.001, Fisher’s exact test). In contrast, nine STs constituted the group 4, and the isolates of this group were mainly from artificial water sources (68.75%, 33/48, P<0.01, Chi-Square test). These results showed distinct phylogenetic patterns between the isolates from the two environments. The topology of the two inferred trees was not congruent since, depending on the concatenated SBT and MLST sequences, most isolates had different relationships with each other (Figs 1 and 2, S1 Table). However, we still found out an accordance between STs and nSTs on their respective trees, although not completely. For example, the isolates A5, A189, and A195 were clustered into a clade in the ST tree (ST1778, ST160 and ST19, group 1 of the ST tree, Fig 2, S1 Table). They were also situated in a clade in the nST tree (nST5 and nST36, group 5 of the nST tree, Fig 1, S1 Table). N71 and N220 were both ST45 isolates. They belonged to nST66 and nST91, and were clustered into a clade in the nST tree (Fig 1). N36, N37, N38, N39, N40, N41, and N43 were both ST739 isolates, but they belonged to nST44, nST45, nST46, nST47, nST48, and nST49, respectively. These twelve isolates and their respective branches were clustered into group 1 of the ST tree (Fig 2), while their respective nST branches distributed among three groups (Fig 1). These results showed different phylogenetic relationships between *L*. *pneumophila* isolates from natural and artificial water sources, demonstrated the partial correspondence of the MLST with SBT, and implied more discriminatory ability of the MLST scheme for environmental *L*. *pneumophila* isolates.

### Recombinations in environmental *L*. *pneumophila* isolates

**Fig 3:**
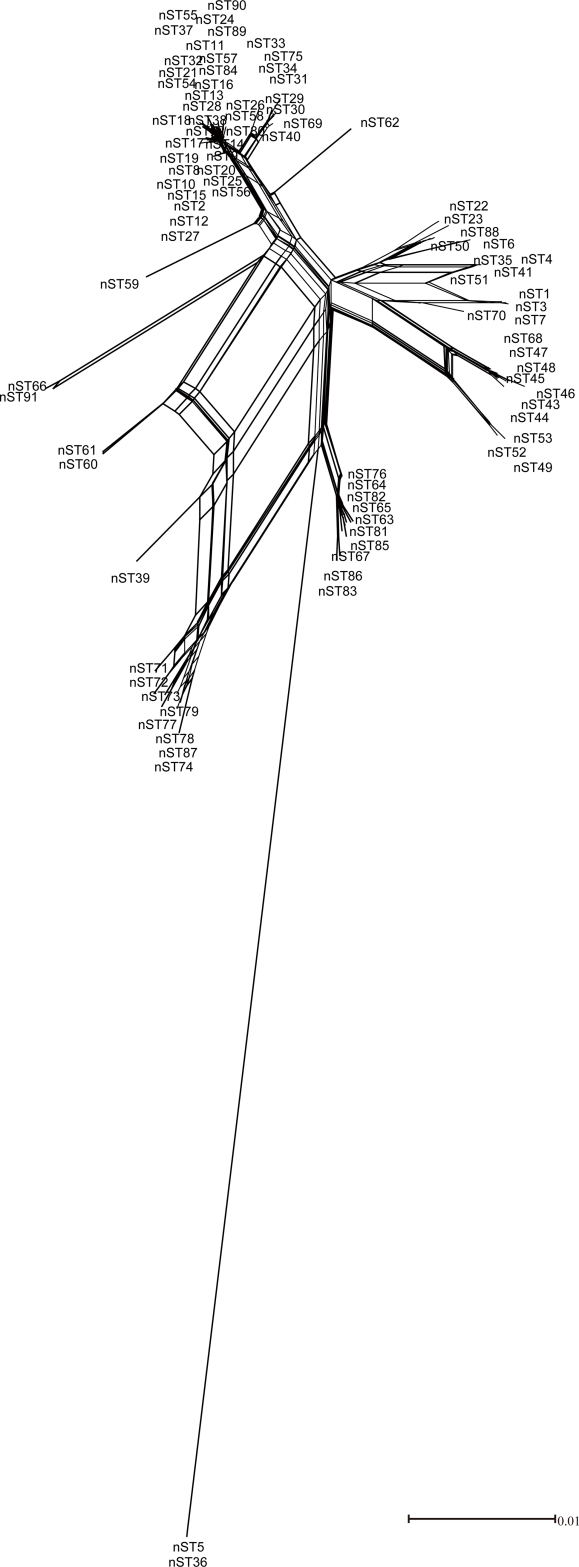
Reticulate network tree by using the neighbor-net algorithm of SplitsTree4 using the five MLST loci concatenated alignments of the 91 nSTs. All internal nodes represent hypothetical ancestral nSTs and edges correspond to reticulate events such as recombinations.

**Fig 4:**
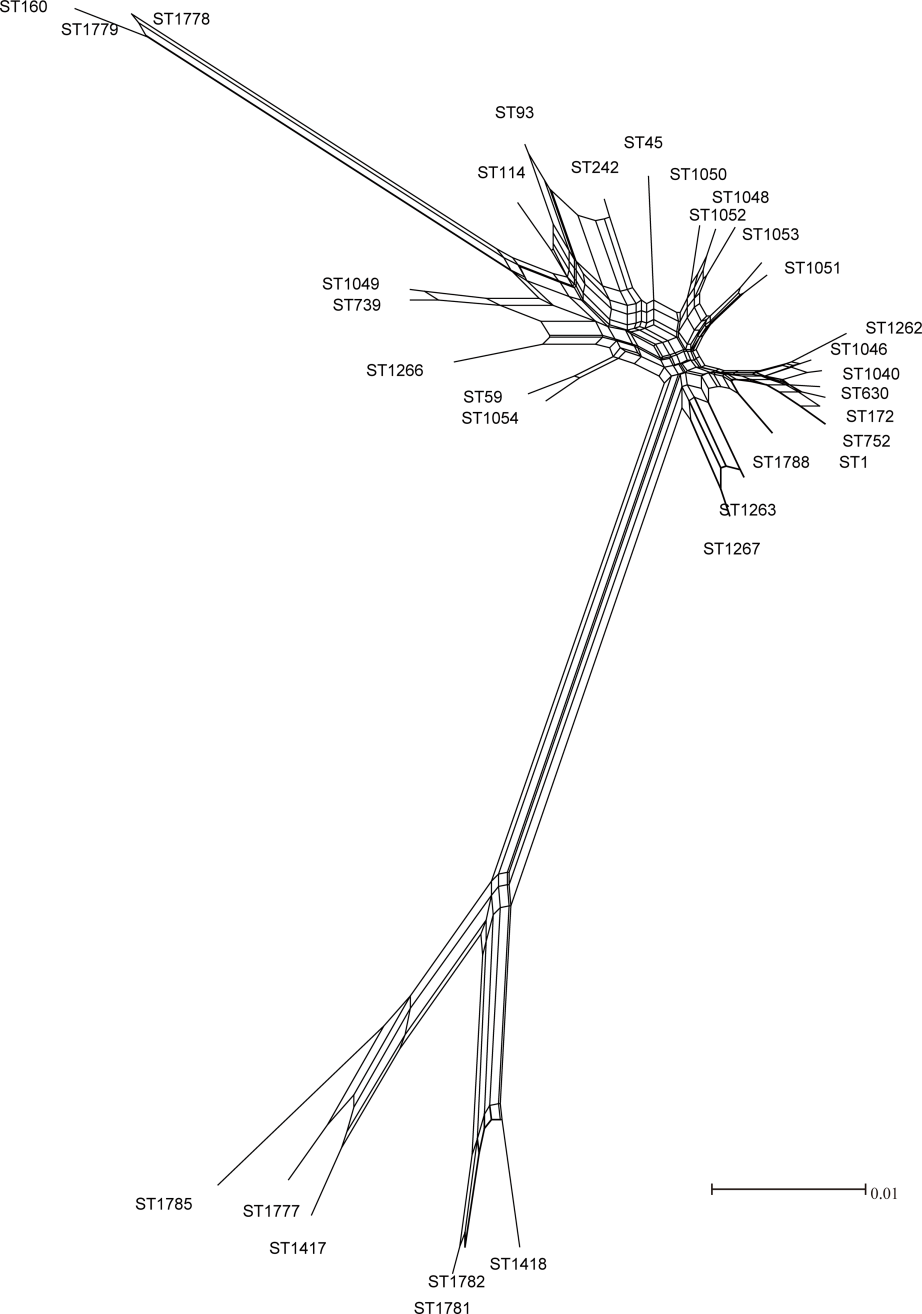
Reticulate network tree by using the neighbor-net algorithm of SplitsTree4 using the seven SBT loci concatenated alignments of the 33 STs. All internal nodes represent hypothetical ancestral STs and edges correspond to reticulate events such as recombinations.

Many studies have reported that recombinations existed in *L*. *pneumophila* isolates. Costa has detected recombinations in *L*. *pneumophila* virulence-related effector *sidJ* within *L*. *pneumophila subsp*. *pneumophila* strains [21]. Recombination is an important mechanism that shaped *L*. *pneumophila* genomes [44]. In this study, the bootstrap values for some branches in the ML trees of STs and nSTs were less than 50%, implying that incongruence phylogeny of the tested nSTs and STs and possible recombination events in the population (Figs 1 and 2) [43]. We obtained reticulate network trees of the concatenated sequences of STs and nSTs by using the neighbor-net algorithm of SplitsTree4 [32] (Version 4.14.4). In the basis of the reticulate tree, a pure clonal population will not have any side edges, while we could find many side edges in reticulate network trees of the 33 STs and 91 nSTs (Figs 3 and 4). This result indicated that recombination events might exist within the population [45]. Thus we tested the intragenic recombinations in the concatenated SBT and MLST sequences separately by using RDP 4. Thirteen PREs among STs, and 14 PREs among nSTs were identified, which were supported by at least two of the six analysis methods (Table 6 and Table 7). Among the 41 resulting recombinant nSTs, three nSTs (nST22, nST23, nST39) were exclusively found in the isolates from artificial water sources, and 38 nSTs were exclusively found in the isolates from natural ones. Similarly, among the 20 recombinant STs, thirteen were exclusively found in natural water sources, and five were exclusively found in the artificial ones. These results together showed a higher frequency of recombinations existed in the isolates from natural water sources, which was consistent with a higher diversity in these isolates.

**Table 6:** Intragenic recombination in the 33 STs by using six different methods implemented in RDP software.

Recombination events	Recombinant STs	Major parent*	Minor parent^#^	Detection methods implemented in RDP software^$^					
				RDP	GENECONV	Bootscan	Maxchi	Chimaera	SiSscan
1	ST1417, ST1781, ST1782, ST1785, ST1777	ST1788	ST160^a^	Y^b^	Y	Y	Y	Y	N^c^
2	ST1418	ST1051	ST160^a^	Y	Y	N	Y	Y	N
3	ST160	ST1778	ST242^a^	Y	Y	N	Y	Y	N
4	ST114, ST59, ST1054	ST1049^a^	ST1263	Y	Y	Y	Y	Y	Y
5	ST93, ST242	ST1052	ST1049^a^	N	N	N	Y	Y	N
6	ST1262, ST1263	ST172	ST1052^a^	Y	Y	Y	Y	Y	N
7	ST1267	ST1051	ST1778^a^	N	Y	N	N	N	Y
8	ST1417	ST1267^a^	ST1050	Y	N	N	Y	N	Y
9	ST1263, ST1417, ST1781	ST752	ST1785^a^	Y	N	N	N	N	Y
10	ST1266	ST1049	ST1051	Y	Y	Y	Y	Y	Y
11	ST1049, ST739	ST45	ST1778	Y	N	N	Y	Y	N
12	ST1051, ST59	ST1053	ST1778	N	Y	N	N	N	Y
13	ST1053, ST1051	ST1050	ST1785	Y	N	N	N	N	Y

* Major parent: parent contributing the larger fraction of the sequence.

# Minor parent: parent ST contributing the smaller fraction of the sequence.

$ Recombination events detected by more than two methods were shown.

a ST used to infer the existence of a missing parental sequence

b Y indicates recombination events were detected by the selected method.

c N indicates recombination events were not detected by the selected method.

**Table 7:** Intragenic recombination in the 91 nSTs by using six different methods implemented in RDP software.

Recombination events	Recombinant nSTs	Major parent*	Minor parent^#^	Detection methods implemented in RDP software^$^					
				RDP	GENECONV	Bootscan	Maxchi	Chimaera	SiSscan
1	nST39	nST6	nST59^a^	Y^b^	Y	Y	Y	Y	Y
2	nST60, nST61	nST59	nST6^a^	Y	Y	N^c^	Y	Y	N
3	nST83, nST63, nST65, nST81, nST76, nST64, nST67, nST85, nST82, nST86	nST72	nST62	Y	Y	N	Y	Y	Y
4	nST22, nST23, nST50, nST51, nST88	nST37	nST87	Y	Y	Y	Y	Y	Y
5	nST71, nST72, nST67, nST73, nST74, nST77, nST78, nST79, nST83, nST86, nST87	nST41	nST33^a^	Y	Y	N	Y	N	Y
6	nST91, nST66	nST41	nST59^a^	Y	Y	N	Y	Y	Y
7	nST59	nST70	nST87	N	Y	N	Y	Y	Y
8	nST70	nST4	nST41^a^	N	N	N	Y	Y	Y
9	nST59	nST39	nST33	Y	Y	Y	Y	N	Y
10	nST41	nST4	nST49^a^	Y	Y	Y	Y	Y	Y
11	nST60, nST61	nST72^a^	nST33	Y	N	Y	Y	N	Y
12	nST46, nST43, nST44, nST45, nST47, nST48, nST49, nST68	nST4^a^	nST33	N	N	N	Y	Y	N
13	nST53, nST52	nST46	nST62^a^	Y	N	N	N	N	Y
14	nST91, nST66	nST87^a^	nST22	N	N	N	Y	N	Y

* Major parent: parent contributing the larger fraction of the sequence.

# Minor parent: parent ST contributing the smaller fraction of the sequence

$ Recombination events detected by more than two methods were shown.

a nST used to infer the existence of a missing parental sequence

b Y indicates recombination events were detected by the selected method

c N indicates recombination events were not detected by the selected method.

Although early analysis based on multilocus enzyme electrophoresis (MLEE) described the population structure of this species as clonal, many recent reports have suggested that recombination also contributed to shaping variation across its genome [21, 44, 46–49]. Coscolla reported that recombinations among *L*. *pneumophila* isolates from natural water sources are common, and not restricted to already described pathogenicity islands or other genome constituents, which provided the genome with high plasticity [46]. Recombinations were also found in outbreak-related *L*. *pneumophil*a isolates [47]. Our results based on nSTs and STs, together with previously reports, supported the notion that *L*. *pneumophila* was undergoing recombinations, especially in those isolates from natural water sources. Recombination was a relevant factor in shaping molecular population genetic structure of this bacterium, and might contribute to the higher diversity of nSTs than that of STs, observed in our environmental isolate collection.

### Five-gene MLST scheme to subtype the major and abundant disease-associated ST1 isolates

**Fig 5:**
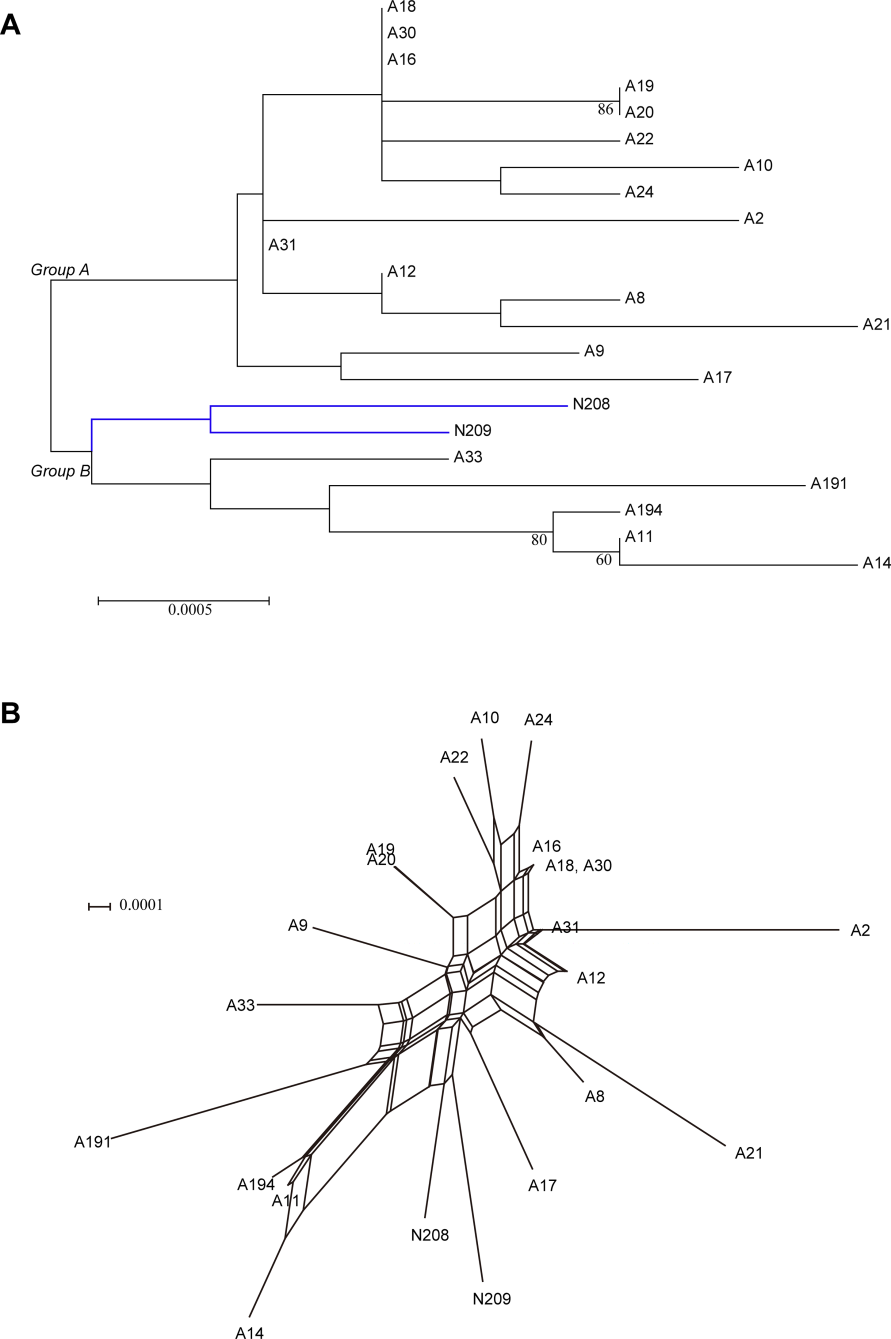
Phylogenetic tree and reticulate network tree of the concatenated MLST sequences (2876bp) for the 22 ST1 isolates. **A.** Phylogenetic tree of the concatenated MLST sequences (2876bp) for the 22 ST1 isolates in this study. Two main groups of these isolates could be found in the tree. The blue branches indicate the natural water isolates (N), which constitute a subgroup. **B.** Reticulate network tree of the concatenated sequences of the MLST loci for the 22 ST1 isolates. Internal nodes and edges exist.

In this study, we obtained 22 ST1 isolates from water sources in Guangdong Province of China. They were mainly from artificial water sources (90.91%, 20/22, Table 2), and could be subtyped into 19 nSTs (S5 Table), indicating extraordinary discrimination of the five-gene MLST for environmental ST1 isolates. Many studies have reported that cgMLST could provide a high resolution in subtyping ST1 isolates [13, 16, 50], but these schemes sequenced thousands of core genes shared by different *L*. *pneumophila* strains. The MLST scheme reported in the present study only sequenced five loci, and the concatenated sequence length was comparable with that of the SBT (2876 bp vs. 2501/2498 bp), but provided a notable resolution. As shown in Fig 5A, the 22 ST1 isolates could be clustered into two main groups (group A and group B). ST1 isolates from natural water sources (N208 and N209) also formed a subgroup. This result suggested that the phylogeny of these two ST1 isolates were closer to each other, and genetic differences might exist between ST1 isolates from natural and artificial water sources. Reticulate network tree of the ST1 isolates showed many side edges, indicating the recombinations of the MLST sequences also exist within these isolates (Fig 5B).

### Five-gene MLST scheme to genotype reference clinical and environmental *L*. *pneumophila* strains

**Fig 6:**
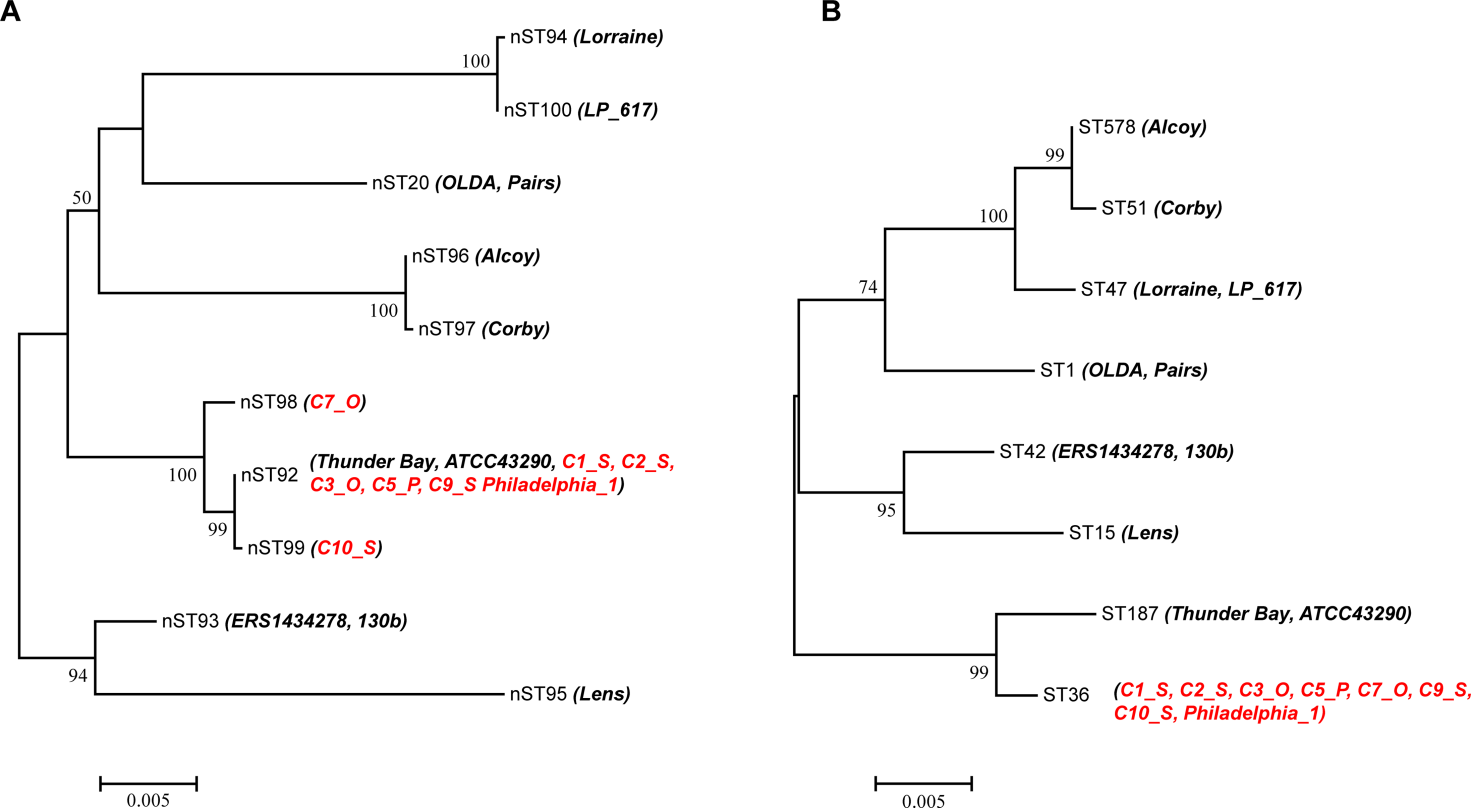
Phylogenetic analysis of the concatenated MLST and SBT sequences for the nSTs and STs of the clinical strains. Bootstrap support values (1000 replicates) for nodes higher than 50% are indicated next to the corresponding node. **A.** ML tree of the concatenated MLST sequences for the 10 nSTs of the 19 clinical strains. The ST36 isolates were marked red. **B.** ML tree of the concatenated SBT sequences for the eight STs of the 19 clinical strains.

As shown in Table 1, the nineteen clinical strains were assigned to 10 nSTs and 8 STs. The IODs of the SBT and MLST for this strain collection were 0.770 and 0.781, respectively, suggesting the MLST scheme was also more discriminatory for clinical strains. The nSTs and STs of the reference clinical strains were not found in our environmental isolate collection except the nST20 and the ST1. The initial LD isolate, Philadelphia-1 is an ST36 (also called Philadelphia sequence type) strain and was discovered on the outbreak of Philadelphia LD in 1976 [51]. After that, many ST36 isolates were found in outbreak investigations and sporadic cases in the USA [52]. ST36 was the most frequent ST that associated with LD outbreak in the USA during 1982 and 2012 [52]. It was also prevalent both in clinical and environmental isolates distributed over 25 countries. The first clinical strain that isolated in Chinese mainland also belonged to ST36 [53]. The MLST scheme could subtype the eight clinical ST36 strains to three nSTs (nST92, nST98, and nST99) (Fig 6A). However, the outbreak ST36 isolates (C3_O, C7_O, and Philadelphia-1) could be subtyped into nST92 and nST98, and the sporadic ST36 isolates (C1_S, C2_S, C9_S, and C10_S) could be subtyped into nST92 and nST99 (Table 1). These clinical ST36 isolates situated in a clade of the ML tree of nSTs (Fig 6A). It was interesting that nST98 was exclusively found in the outbreak ST36 isolate (C7_O), while nST99 was exclusively found in the sporadic ST36 isolate (C10_S), and the phylogeny of the two nSTs was distinctive (Fig 6A). A nine-nucleotide difference in the *trpA* locus was found between nST98 and nST92, while only a single nucleotide difference in the *icmK* locus was found between nST99 and nST92, and these nucleotide differences were all found between nST98 and nST99 (data not shown). This would illustrate that some sporadic isolates and outbreak isolates were genetically different. We found two ST187 strains; Thunder Bay and ATCC43290 shared the nST92 with the clinical ST36 isolates, including C1_S, C2_S, C3_O, C5_P, C7_O, C9_S, C10_S, and Philadelphia_1 (Fig 6B). The allelic profiles of ST36 and ST187 were 3, 4, 1, 1, 14, 9, 1 and 3, 10, 1, 28, 14, 9, 3, respectively. There were three loci (*pilE*, *mip*, and *neuA*) differences between the two STs, and contributed to 18 nucleotide differences, implying incongruous phylogenetic relationships between the SBT and MLST sequences in the clinical isolates, which have also been observed in our environmental isolate collection (Figs 1 and 2). We also used ten additional reference environmental ST36 strains to study the discriminatory ability of the MLST for isolates belonging to a same ST (ST36) but from different sources (clinical and environmental) (Table 1). The ten environmental ST36 isolates could also be subtyped into three nSTs (nST3, nST92, and nST101). NST92 was found in both clinical and environmental ST36 isolates, and was the most prevalent nSTs of the eighteen ST36 isolates (13/18, 72.22%). NST3 and nST101 were exclusively found in environmental isolates, while nST98 and nST99 were exclusively found in clinical isolates, indicating different distribution patterns of nSTs between environmental and clinical ST36 isolates. Phylogenetic analysis of these ST36 isolates showed two main groups. NST98 (C7_O) was situated on its own distinct branch, separated from other four nSTs (nST3, nST92, nST99, and nST101) (S1 Fig). These results suggested that the MLST scheme could also subtype the prevalent ST36 isolates, and the phylogenetic relationships among ST36 isolates from clinical and environmental sources might be different, which was supported by Mercante and colleague [51]. ST47 was most frequently isolated from patients in many countries such as Netherlands and France [54, 55]. In this study, two ST47 strains, Lorraine and LP_617 could be subtyped into two nSTs: nST94 and nST100. The phylogenetic tree of the concatenated MLST sequences showed these isolates were closely related to each other and clustered into a clade (Fig 6A). We have found similar phylogenetic relationship between Lorraine and LP_617 in a pilot study of rapid whole-genome sequencing for the investigation of a *Legionella* outbreak, in which single-nucleotide polymorphism (SNP)-based (also known as mapping-based) approach was performed, and it showed that LP_617 was only 56 SNPs different from Lorraine in the genome, and thus the two ST47 strains could be distinguished [56]. This fact highlighted the possibility that the MLST scheme also had discriminatory ability for some strains with very small genetic differences. As we know, traditional background mutation, gene deletion, episomal loss/acquisition, and horizontal gene transfer have led to varying degrees of genetic divergence in a related subpopulation of *L*. *pneumophila* [57]. Furthermore, we also found more PREs of nSTs than those of STs in our environmental isolates (Table 5 and Table 6). We supposed these factors might contribute to the accelerated evolution of the MLST loci compared with the SBT loci and lead to the generation of new allelic profiles of nSTs, as it was well believed that clinical *L*. *pneumophila* was a small specific subset of all genotypes existing in nature, perhaps representing an especially adapted group of clones [39].

Two ST1 strains, OLDA and Paris were both assigned to nST20. One environmental ST36 isolate (E8_O), which was proved to be associated with LD outbreak was assigned to nST3. In our environmental collection of *L*. *pneumophila*, an ST1 isolate (A31) and an ST630 isolate (A23) were both nST20, and an ST242 (A3) isolate was nST3. However, most of our environmental isolates typed as ST1 were characterized by different nSTs, and only nST20 and nST3 could be found in the clinical strains or strains associated with LD outbreak. Furthermore, we found higher discriminatory power of the MLST for the environmental isolates than for the clinical isolates. In light of these findings, the role of environmental sources as a potential reservoir of distinct pathogens could be reinforced [58]. ML trees of the ten nSTs and eight STs of the clinical isolates both showed two main groups. However, the isolates constituted these groups were different. NST93 (ERS1434278 and 130b) and nST95 (Lens) constituted a distinct clade in the ML tree of nSTs, while ST187 (Thunder Bay and ATCC43290) and ST36 (C1_S, C2_S, C3_O, C5_P, C7_O, C9_S, C10_S, and Philadelphia_1) constituted a distinct clade in the ML tree of STs (Fig 6). We also found a relatively longer phylogenetic distance of the MLST sequences than that of the SBT sequences within the clinical isolates. For example, the phylogenetic distance between ERS1434278 and Lens of the nST tree was longer than that of the ST tree (Fig 6). These results together suggested that the MLST scheme was a more discriminatory means for epidemiological investigation of clinical and environmental *L*. *pneumophila* isolates. It is well known that the major advantage of SBT has been the ease of exchanging data between different laboratories, but the evidence of a large proportion of cases is caused by a small number of common STs (e.g., ST1 and ST47) indicated this scheme lacked discriminatory power [13]. Thus the five-gene MLST scheme we proposed here might be used as a supplementary method for epidemiological investigation of *L*. *pneumophila*.

## Conclusions

Although there have been many studies probing new typing methods for *L*. *pneumophila*, such as SNP-based approach [56], whole-genome mapping (WGM) [17], cgMLST [13, 50] and rMLST [59], these schemes required to sequence a great many of gene loci, and the cost and bioinformatics infrastructure might be issues in some laboratories. In this study, we reported a five-gene MLST scheme for genotyping of *L*. *pneumophila* isolates from environmental water samples and clinical samples, and compared with the SBT. Our results showed higher discriminatory power of the MLST for our environmental isolate collection. We have described the differences in ST and nST distributions and diversities of *L*. *pneumophila* isolates from natural and artificial water sources in Guangdong province of China. We found intragenic recombination might be one of an important mechanism that contributed to higher discrimination of MLST, and higher diversities of STs and nSTs in natural water isolates. The MLST scheme also showed an extraordinary resolution in subtyping environmental ST1 isolates and high discriminatory power in genotyping clinical *L*. *pneumophila* strains. In addition, the MLST scheme could subtype the clinical isolates belonging to prevalent STs (ST36 and ST47). We found different distribution patterns of nSTs between environmental and clinical ST36 isolates, and between the outbreak clinical ST36 isolates and the sporadic clinical ST36 isolates. These results together suggested that the MLST scheme could be used as part of a typing scheme that increased discrimination when necessary.

## Supporting information

S1 Table *L*. *pneumophila* environmental isolates information. (DOCX)

S2 Table Sequence variation of the MLST loci and primers for the MLST scheme. (DOCX)

S3 Table Number of sequence types and IODs of the environmental isolates achieved by the SBT and the five-gene MLST. (DOCX)

S4 Table Distribution of environmental *L*. *pneumophila* isolates in each group of ST or nST. (DOCX)

S5 Table Environmental ST1 isolates information. (DOCX)

S1 Fig Phylogeny of clinical and environmental ST36 isolates based on the concatenated MLST sequences (2876bp). The clinical isolates were marked red, while the environmental isolates were marked blue.
(TIF)

## References

[1] Fliermans CB, Cherry WB, Orrison LH, Smith SJ, Tison DL, Pope DH. Ecological distribution of Legionella pneumophila. Appl Environ Microbiol. 1981;41(1):9–16. ; PubMed Central PMCID: PMC243633. 7013702 10.1128/aem.41.1.9-16.1981 PMC243633

[2] Fields BS, Benson RF, Besser RE. Legionella and Legionnaires' disease: 25 years of investigation. Clin Microbiol Rev. 2002;15(3):506–26. doi: 10.1128/CMR.15.3.506-526.2002 ; PubMed Central PMCID: PMC118082. 12097254 10.1128/CMR.15.3.506-526.2002 PMC118082

[3] Gomez-Valero L, Rusniok C, Rolando M, Neou M, Dervins-Ravault D, Demirtas J, et al Comparative analyses of Legionella species identifies genetic features of strains causing Legionnaires' disease. Genome Biol. 2014;15(11):505 doi: 10.1186/s13059-014-0505-0 ; PubMed Central PMCID: PMC4256840. 25370836 10.1186/s13059-014-0505-0 PMC4256840

[4] Furuhata K, Ishizaki N, Umekawa N, Nishizima M, Fukuyama M. Pulsed-Field Gel Electrophoresis (PFGE) pattern analysis and chlorine-resistance of Legionella pneumophila isolated from hot spring water samples. Biocontrol Sci. 2014;19(1):33–8. . 24670616 10.4265/bio.19.33

[5] Yuan M, Yuan YM, Mu-Hua YU. Molecular typing methods for Legionella pneumophila detection. Chinese Journal of Public Health. 2010.

[6] Guo YM, Zhou JK, Zhang HX, Qin LY, Zhao D, Hui-Dong JU, et al Comparison among SBT,PFGE,AFLP Molecular Typing Methods of Legionella pneumophila. J Microbiol. 2014;34(1):72–7.

[7] Ginevra C. Legionella pneumophila Typing 2013 221–7 p.

[8] Lepeuple AS, Jovic M, de Roubin MR. Molecular typing of the Legionella pneumophila population isolated from several locations in a contaminated water network. Water Science & Technology. 2004;50(1):281–5. 15318523

[9] Gaia V, Fry NK, Afshar B, Luck PC, Meugnier H, Etienne J, et al Consensus sequence-based scheme for epidemiological typing of clinical and environmental isolates of Legionella pneumophila. J Clin Microbiol. 2005;43(5):2047–52. doi: 10.1128/JCM.43.5.2047-2052.2005 ; PubMed Central PMCID: PMC1153775. 15872220 10.1128/JCM.43.5.2047-2052.2005 PMC1153775

[10] Ratzow S, Gaia V, Helbig JH, Fry NK, Luck PC. Addition of neuA, the gene encoding N-acylneuraminate cytidylyl transferase, increases the discriminatory ability of the consensus sequence-based scheme for typing Legionella pneumophila serogroup 1 strains. J Clin Microbiol. 2007;45(6):1965–8. doi: 10.1128/JCM.00261-07 ; PubMed Central PMCID: PMC1933043. 17409215 10.1128/JCM.00261-07 PMC1933043

[11] Urwin R, Maiden MC. Multi-locus sequence typing: a tool for global epidemiology. Trends Microbiol. 2003;11(10):479–87. . 14557031 10.1016/j.tim.2003.08.006

[12] Gaia V, Fry NK, Harrison TG, Peduzzi R. Sequence-Based Typing of Legionella pneumophila Serogroup 1 Offers the Potential for True Portability in Legionellosis Outbreak Investigation. 2003;41(7):2932–9. 10.1128/JCM.41.7.2932-2939.2003 PMC165343 12843023

[13] David S, Mentasti M, Tewolde R, Aslett M, Harris SR, Afshar B, et al Evaluation of an Optimal Epidemiological Typing Scheme for Legionella pneumophila with Whole-Genome Sequence Data Using Validation Guidelines. J Clin Microbiol. 2016;54(8):2135–48. doi: 10.1128/JCM.00432-16 ; PubMed Central PMCID: PMCPMC4963484. 27280420 10.1128/JCM.00432-16 PMC4963484

[14] Ginevra C, Forey F, Campese C, Reyrolle M, Che D, Etienne J, et al Lorraine strain of Legionella pneumophila serogroup 1, France. Emerg Infect Dis. 2008;14(4):673–5. doi: 10.3201/eid1404.070961 ; PubMed Central PMCID: PMC2570941. 18394295 10.3201/eid1404.070961 PMC2570941

[15] Mercante JW, Morrison SS, Desai HP, Raphael BH, Winchell JM. Genomic Analysis Reveals Novel Diversity among the 1976 Philadelphia Legionnaires’ Disease Outbreak Isolates and Additional ST36 Strains. Plos One. 2016;11(9). 10.1371/journal.pone.0164074 PMC5042515 27684472

[16] Moran-Gilad J, Prior K, Yakunin E, Harrison TG, Underwood A, Lazarovitch T, et al Design and application of a core genome multilocus sequence typing scheme for investigation of Legionnaires' disease incidents. Euro Surveill. 2015;20(28). . 26212142 10.2807/1560-7917.es2015.20.28.21186

[17] Bosch T, Euser SM, Landman F, Bruin JP, EP IJ, den Boer JW, et al Whole-Genome Mapping as a Novel High-Resolution Typing Tool for Legionella pneumophila. J Clin Microbiol. 2015;53(10):3234–8. doi: 10.1128/JCM.01369-15 ; PubMed Central PMCID: PMCPMC4572561. 26202110 10.1128/JCM.01369-15 PMC4572561

[18] Graham RM, Doyle CJ, Jennison AV. Real-time investigation of a Legionella pneumophila outbreak using whole genome sequencing. Epidemiol Infect. 2014;142(11):2347–51. doi: 10.1017/S0950268814000375 . 24576553 10.1017/S0950268814000375 PMC9151283

[19] Lau HY, Ashbolt NJ. The role of biofilms and protozoa in Legionella pathogenesis: implications for drinking water. J Appl Microbiol. 2009;107(2):368–78. doi: 10.1111/j.1365-2672.2009.04208.x . 19302312 10.1111/j.1365-2672.2009.04208.x

[20] Escoll P, Rolando M, Gomez-Valero L, Buchrieser C. From amoeba to macrophages: exploring the molecular mechanisms of Legionella pneumophila infection in both hosts. Curr Top Microbiol Immunol. 2013;376:1–34. doi: 10.1007/82_2013_351 . 23949285 10.1007/82_2013_351

[21] Costa J, Teixeira PG, d'Avo AF, Junior CS, Verissimo A. Intragenic recombination has a critical role on the evolution of Legionella pneumophila virulence-related effector sidJ. PLoS One. 2014;9(10):e109840 doi: 10.1371/journal.pone.0109840 ; PubMed Central PMCID: PMCPMC4192588. 25299187 10.1371/journal.pone.0109840 PMC4192588

[22] Borges V, Nunes A, Sampaio DA, Vieira L, Machado J, Simoes MJ, et al Legionella pneumophila strain associated with the first evidence of person-to-person transmission of Legionnaires' disease: a unique mosaic genetic backbone. Sci Rep. 2016;6:26261 doi: 10.1038/srep26261 ; PubMed Central PMCID: PMCPMC4872527. 27196677 10.1038/srep26261 PMC4872527

[23] Zhan XY, Zhu QY. Molecular evolution of virulence genes and non-virulence genes in clinical, natural and artificial environmental Legionella pneumophila isolates. PeerJ. 2017;5:e4114 doi: 10.7717/peerj.4114 29226035 10.7717/peerj.4114 PMC5719964

[24] Zhan XY, Hu CH, Zhu QY. Different distribution patterns of ten virulence genes in Legionella reference strains and strains isolated from environmental water and patients. Arch Microbiol. 2016;198(3):241–50. doi: 10.1007/s00203-015-1186-0 . 26757724 10.1007/s00203-015-1186-0

[25] Hunter PR, Gaston MA. Numerical index of the discriminatory ability of typing systems: an application of Simpson's index of diversity. J Clin Microbiol. 1988;26(11):2465–6. ; PubMed Central PMCID: PMCPMC266921. 3069867 10.1128/jcm.26.11.2465-2466.1988 PMC266921

[26] Rozas J. DNA sequence polymorphism analysis using DnaSP. Methods Mol Biol. 2009;537:337–50. doi: 10.1007/978-1-59745-251-9_17 . 19378153 10.1007/978-1-59745-251-9_17

[27] Librado P, Rozas J. DnaSP v5: a software for comprehensive analysis of DNA polymorphism data. Bioinformatics. 2009;25(11):1451–2. doi: 10.1093/bioinformatics/btp187 . 19346325 10.1093/bioinformatics/btp187

[28] Excoffier L, Lischer HE. Arlequin suite ver 3.5: a new series of programs to perform population genetics analyses under Linux and Windows. Mol Ecol Resour. 2010;10(3):564–7. doi: 10.1111/j.1755-0998.2010.02847.x . 21565059 10.1111/j.1755-0998.2010.02847.x

[29] Kumar S, Stecher G, Tamura K. MEGA7: Molecular Evolutionary Genetics Analysis Version 7.0 for Bigger Datasets. Mol Biol Evol. 2016;33(7):1870–4. doi: 10.1093/molbev/msw054 . 27004904 10.1093/molbev/msw054 PMC8210823

[30] Kimura M. A simple method for estimating evolutionary rates of base substitutions through comparative studies of nucleotide sequences. J Mol Evol. 1980;16(2):111–20. . 7463489 10.1007/BF01731581

[31] Bryant D, Moulton V. Neighbor-net: an agglomerative method for the construction of phylogenetic networks. Mol Biol Evol. 2004;21(2):255–65. doi: 10.1093/molbev/msh018 . 14660700 10.1093/molbev/msh018

[32] Huson DH, Bryant D. Application of phylogenetic networks in evolutionary studies. Mol Biol Evol. 2006;23(2):254–67. doi: 10.1093/molbev/msj030 . 16221896 10.1093/molbev/msj030

[33] Martin DP, Murrell B, Khoosal A, Muhire B. Detecting and Analyzing Genetic Recombination Using RDP4. Methods Mol Biol. 2017;1525:433–60. doi: 10.1007/978-1-4939-6622-6_17 . 27896731 10.1007/978-1-4939-6622-6_17

[34] Martin D, Rybicki E. RDP: detection of recombination amongst aligned sequences. Bioinformatics. 2000;16(6):562–3. . 10980155 10.1093/bioinformatics/16.6.562

[35] Martin DP, Posada D, Crandall KA, Williamson C. A modified bootscan algorithm for automated identification of recombinant sequences and recombination breakpoints. AIDS Res Hum Retroviruses. 2005;21(1):98–102. doi: 10.1089/aid.2005.21.98 . 15665649 10.1089/aid.2005.21.98

[36] Smith JM. Analyzing the mosaic structure of genes. J Mol Evol. 1992;34(2):126–9. . 1556748 10.1007/BF00182389

[37] Posada D. Evaluation of methods for detecting recombination from DNA sequences: empirical data. Mol Biol Evol. 2002;19(5):708–17. doi: 10.1093/oxfordjournals.molbev.a004129 . 11961104 10.1093/oxfordjournals.molbev.a004129

[38] Gibbs MJ, Armstrong JS, Gibbs AJ. Sister-scanning: a Monte Carlo procedure for assessing signals in recombinant sequences. Bioinformatics. 2000;16(7):573–82. . 11038328 10.1093/bioinformatics/16.7.573

[39] Coscolla M, Gonzalez-Candelas F. Comparison of clinical and environmental samples of Legionella pneumophila at the nucleotide sequence level. Infect Genet Evol. 2009;9(5):882–8. doi: 10.1016/j.meegid.2009.05.013 . 19465160 10.1016/j.meegid.2009.05.013

[40] Pancer K. Sequence-based typing of Legionella pneumophila strains isolated from hospital water distribution systems as a complementary element of risk assessment of legionellosis in Poland. Ann Agric Environ Med. 2013;20(3):436–40. . 24069845

[41] Zhang L, Li Y, Wang X, Shangguan Z, Zhou H, Wu Y, et al High Prevalence and Genetic Polymorphisms of Legionella in Natural and Man-Made Aquatic Environments in Wenzhou, China. Int J Environ Res Public Health. 2017;14(3). doi: 10.3390/ijerph14030222 ; PubMed Central PMCID: PMCPMC5369058. 28245548 10.3390/ijerph14030222 PMC5369058

[42] Reimer AR, Au S, Schindle S, Bernard KA. Legionella pneumophila monoclonal antibody subgroups and DNA sequence types isolated in Canada between 1981 and 2009: Laboratory Component of National Surveillance. Eur J Clin Microbiol Infect Dis. 2010;29(2):191–205. doi: 10.1007/s10096-009-0840-3 . 19960359 10.1007/s10096-009-0840-3

[43] Guo J, Liang T, Hu C, Lv R, Yang X, Cui Y, et al Sequence types diversity of Legionella pneumophila isolates from environmental water sources in Guangzhou and Jiangmen, China. Infect Genet Evol. 2015;29:35–41. doi: 10.1016/j.meegid.2014.10.023 . 25445655 10.1016/j.meegid.2014.10.023

[44] Gomez-Valero L, Rusniok C, Jarraud S, Vacherie B, Rouy Z, Barbe V, et al Extensive recombination events and horizontal gene transfer shaped the Legionella pneumophila genomes. BMC Genomics. 2011;12:536 doi: 10.1186/1471-2164-12-536 ; PubMed Central PMCID: PMC3218107. 22044686 10.1186/1471-2164-12-536 PMC3218107

[45] Morrison DA. Networks in phylogenetic analysis: new tools for population biology. Int J Parasitol. 2005;35(5):567–82. doi: 10.1016/j.ijpara.2005.02.007 . 15826648 10.1016/j.ijpara.2005.02.007

[46] Coscolla M, Gonzalez-Candelas F. Population structure and recombination in environmental isolates of Legionella pneumophila. Environ Microbiol. 2007;9(3):643–56. doi: 10.1111/j.1462-2920.2006.01184.x . 17298365 10.1111/j.1462-2920.2006.01184.x

[47] Sanchez-Buso L, Comas I, Jorques G, Gonzalez-Candelas F. Recombination drives genome evolution in outbreak-related Legionella pneumophila isolates. Nat Genet. 2014;46(11):1205–11. doi: 10.1038/ng.3114 . 25282102 10.1038/ng.3114

[48] Visca P, D'Arezzo S, Ramisse F, Gelfand Y, Benson G, Vergnaud G, et al Investigation of the population structure of Legionella pneumophila by analysis of tandem repeat copy number and internal sequence variation. Microbiology+. 2011;157(Pt 9):2582–94. doi: 10.1099/mic.0.047258-0 . 21622529 10.1099/mic.0.047258-0

[49] Costa J, d'Avo AF, da Costa MS, Verissimo A. Molecular evolution of key genes for type II secretion in Legionella pneumophila. Environ Microbiol. 2012;14(8):2017–33. doi: 10.1111/j.1462-2920.2011.02646.x . 22118294 10.1111/j.1462-2920.2011.02646.x

[50] Qin T, Zhang W, Liu W, Zhou H, Ren H, Shao Z, et al Population structure and minimum core genome typing of Legionella pneumophila. Sci Rep. 2016;6:21356 doi: 10.1038/srep21356 ; PubMed Central PMCID: PMCPMC4766850. 26888563 10.1038/srep21356 PMC4766850

[51] Mercante JW, Morrison SS, Desai HP, Raphael BH, Winchell JM. Genomic Analysis Reveals Novel Diversity among the 1976 Philadelphia Legionnaires' Disease Outbreak Isolates and Additional ST36 Strains. PLoS One. 2016;11(9):e0164074 doi: 10.1371/journal.pone.0164074 ; PubMed Central PMCID: PMCPMC5042515. 27684472 10.1371/journal.pone.0164074 PMC5042515

[52] Kozak-Muiznieks NA, Lucas CE, Brown E, Pondo T, Jr TT, Frace M, et al Prevalence of sequence types among clinical and environmental isolates of Legionella pneumophila serogroup 1 in the United States from 1982 to 2012. J Clin Microbiol. 2014;52(1):201–11. doi: 10.1128/JCM.01973-13 24197883 10.1128/JCM.01973-13 PMC3911437

[53] Qin T. Liver cirrhosis as a predisposing condition for Legionnaires' disease: a report of four laboratory-confirmed cases from China. J Med Microbiol. 2012;61(Pt 7):1023 doi: 10.1099/jmm.0.040170-0 22466030 10.1099/jmm.0.040170-0

[54] Den Boer JW, Euser SM, Brandsema P, Reijnen L, Bruin JP. Results from the National Legionella Outbreak Detection Program, the Netherlands, 2002–2012. Emerg Infect Dis. 2015;21(7):1167–73. doi: 10.3201/eid2107.141130 ; PubMed Central PMCID: PMCPMC4480379. 26079594 10.3201/eid2107.141130 PMC4480379

[55] Campese C, Bitar D, Jarraud S, Maine C, Forey F, Etienne J, et al Progress in the surveillance and control of Legionella infection in France, 1998–2008. Int J Infect Dis. 2011;15(1):e30–7. doi: 10.1016/j.ijid.2010.09.007 . 21109475 10.1016/j.ijid.2010.09.007

[56] Reuter S, Harrison TG, Koser CU, Ellington MJ, Smith GP, Parkhill J, et al A pilot study of rapid whole-genome sequencing for the investigation of a Legionella outbreak. BMJ Open. 2013;3(1). doi: 10.1136/bmjopen-2012-002175 ; PubMed Central PMCID: PMC3553392. 23306006 10.1136/bmjopen-2012-002175 PMC3553392

[57] de Felipe KS, Pampou S, Jovanovic OS, Pericone CD, Ye SF, Kalachikov S, et al Evidence for acquisition of Legionella type IV secretion substrates via interdomain horizontal gene transfer. J Bacteriol. 2005;187(22):7716–26. doi: 10.1128/JB.187.22.7716-7726.2005 ; PubMed Central PMCID: PMC1280299. 16267296 10.1128/JB.187.22.7716-7726.2005 PMC1280299

[58] McAdam PR, Vander Broek CW, Lindsay DS, Ward MJ, Hanson MF, Gillies M, et al Gene flow in environmental Legionella pneumophila leads to genetic and pathogenic heterogeneity within a Legionnaires' disease outbreak. Genome Biol. 2014;15(11):504 doi: 10.1186/s13059-014-0504-1 ; PubMed Central PMCID: PMC4256819. 25370747 10.1186/s13059-014-0504-1 PMC4256819

[59] Jolley KA, Bliss CM, Bennett JS, Bratcher HB, Brehony C, Colles FM, et al Ribosomal multilocus sequence typing: universal characterization of bacteria from domain to strain. Microbiology (Reading, England). 2012;158(4):1005–15. 10.1099/mic.0.055459-0 PMC3492749 22282518

