# Supplementary material for: Molecular typing of Legionella pneumophila isolates from environmental water samples and clinical samples using a five-gene sequence typing and standard Sequence-Based Typing
Source: PLoS One. 2018 Feb 1;13(2):e0190986. doi: 10.1371/journal.pone.0190986 (PMC5794064; doi:10.1371/journal.pone.0190986)
Supplement: S1 Table — (DOCX) [file pone.0190986.s001.docx]

**S1 Table. *L. pneumophila* environmental isolates information.**

| Strain designation | Source nature | | *Geographic location | Geographic coordinate | Collection date | ST | nST |
| --- | --- | --- | --- | --- | --- | --- | --- |
| **Artificial isolates (n=51)** | | | | | | | |
| A1 | ACC# | | Jiangmen: Tianyue hotel | 22.5237, 113.0464 | 2004-07-30 | 630 | 1 |
| A2 | ACC | | Jiangmen: Tianyue hotel | 22.5237, 113.0464 | 2004-07-30 | 1 | 2 |
| A3 | ACC | | Jiangmen: Tianyue hotel | 22.5237, 113.0464 | 2004-07-30 | 242 | 3 |
| A4 | ACC | | Jiangmen: Tianyue hotel | 22.5237, 113.0464 | 2004-07-30 | 242 | 4 |
| A5 | ACC | | Jiangmen: Tianyue hotel | 22.5237, 113.0464 | 2004-07-30 | 1778 | 5 |
| A6 | ACC | | Jiangmen: Tianyue hotel | 22.5237, 113.0464 | 2004-07-30 | 1417 | 6 |
| A7 | ACC | | Jiangmen: Tianyue hotel | 22.5237, 113.0464 | 2004-07-30 | 242 | 7 |
| A8 | ACC | | Jiangmen: Tianyue hotel | 22.5237, 113.0464 | 2006-06-15 | 1 | 8 |
| A9 | ACC | | Jiangmen: Tianyue hotel | 22.5237, 113.0464 | 2006-06-15 | 1 | 9 |
| A10 | ACC | | Jiangmen: Tianyue hotel | 22.5237, 113.0464 | 2006-06-15 | 1 | 10 |
| A11 | ACC | | Jiangmen: Tianyue hotel | 22.5237, 113.0464 | 2006-06-15 | 1 | 11 |
| A12 | ACC | | Jiangmen: Tianyue hotel | 22.5237, 113.0464 | 2006-06-15 | 1 | 12 |
| A14 | ACC | | Jiangmen: Tianyue hotel | 22.5237, 113.0464 | 2006-06-15 | 1 | 13 |
| A15 | ACC | | Jiangmen: Tianyue hotel | 22.5237, 113.0464 | 2006-06-15 | 1048 | 14 |
| A16 | ACC | | Jiangmen: Tianyue hotel | 22.5237, 113.0464 | 2006-06-15 | 1 | 15 |
| A17 | ACC | | Jiangmen: Tianyue hotel | 22.5237, 113.0464 | 2006-06-15 | 1 | 16 |
| A18 | ACC | | Jiangmen: Tianyue hotel | 22.5237, 113.0464 | 2006-06-15 | 1 | 15 |
| A19 | ACC | | Jiangmen: Tianyue hotel | 22.5237, 113.0464 | 2006-06-15 | 1 | 17 |
| A20 | ACC | | Jiangmen: Tianyue hotel | 22.5237, 113.0464 | 2006-06-15 | 1 | 17 |
| A21 | ACC | | Guangzhou: Zijinyuan hotel | 23.0981, 113.2981 | 2003-10-01 | 1 | 18 |
| A22 | ACC | | Guangzhou: Zijinyuan hotel | 23.0981, 113.2981 | 2003-10-01 | 1 | 19 |
| A23 | ACC | | Guangzhou: Zijinyuan hotel | 23.0981, 113.2981 | 2003-10-01 | 630 | 20 |
| A24 | ACC | | Guangzhou: Zijinyuan hotel | 23.0981, 113.2981 | 2003-10-01 | 1 | 21 |
| A25 | ACC | | Guangzhou: Zijinyuan hotel | 23.0981, 113.2981 | 2003-10-01 | 59 | 22 |
| A26 | ACC | | Guangzhou: Zijinyuan hotel | 23.0981, 113.2981 | 2003-10-01 | 59 | 23 |
| A27 | ACC | | Guangzhou: Zijinyuan hotel | 23.0981, 113.2981 | 2003-10-01 | 1262 | 24 |
| A28 | ACC | | Guangzhou: Zijinyuan hotel | 23.0981, 113.2981 | 2003-10-01 | 1040 | 25 |
| A29 | ACC | | Guangzhou: Zijinyuan hotel | 23.0981, 113.2981 | 2003-10-01 | 1046 | 26 |
| A30 | ACC | | Guangzhou: Zijinyuan hotel | 23.0981, 113.2981 | 2003-10-01 | 1 | 15 |
| A31 | ACC | | Guangzhou: Zijinyuan hotel | 23.0981, 113.2981 | 2003-10-01 | 1 | 20 |
| A32 | ACC | | Guangzhou: Zijinyuan hotel | 23.0981, 113.2981 | 2003-10-01 | 172 | 27 |
| A33 | ACC | | Guangzhou: Zijinyuan hotel | 23.0981, 113.2981 | 2003-10-01 | 1 | 28 |
| A171 | ACC | | Jiangmen: Xinhui Yinbing Hotel | 22.5262, 113.0391 | 2007-09-01 | 752 | 29 |
| A172 | ACC | | Jiangmen: Xinhui Yinbing Hotel | 22.5262, 113.0391 | 2007-09-01 | 752 | 30 |
| A173 | ACC | | Jiangmen: Xinhui Yinbing Hotel | 22.5262, 113.0391 | 2007-09-01 | 752 | 31 |
| A174 | ACC | | Jiangmen: Xinhui Yinbing Hotel | 22.5262, 113.0391 | 2007-09-01 | 1777 | 32 |
| A175 | ACC | | Jiangmen: Xinhui Yinbing Hotel | 22.5262, 113.0391 | 2007-09-01 | 752 | 33 |
| A176 | ACC | | Jiangmen: Xinhui Yinbing Hotel | 22.5262, 113.0391 | 2007-09-01 | 752 | 34 |
| A180 | ACC | | Jiangmen: Xinhui Yinbing Hotel | 22.5262, 113.0391 | 2007-09-01 | 1417 | 35 |
| A181 | ACC | | Jiangmen: Xinhui Yinbing Hotel | 22.5262, 113.0391 | 2007-09-01 | 1417 | 35 |
| A189 | ACC | | Jiangmen: Xinhui Gangzhou Hotel | 22.5289, 113.0347 | 2007-09-01 | 160 | 36 |
| A191 | ACC | | Jiangmen: Xinhui Gangzhou Hotel | 22.5289, 113.0347 | 2007-09-01 | 1 | 37 |
| A194 | ACC | | Jiangmen: Xinhui Gangzhou Hotel | 22.5289, 113.0347 | 2007-09-01 | 1 | 38 |
| A195 | ACC | | Jiangmen: Xinhui Gangzhou Hotel | 22.5289, 113.0347 | 2007-09-01 | 1779 | 5 |
| A196 | ACC | | Jiangmen: Xinhui Gangzhou Hotel | 22.5289, 113.0347 | 2007-09-01 | 1054 | 39 |
| A197 | ACC | | Jiangmen: Xinhui Gangzhou Hotel | 22.5289, 113.0347 | 2007-09-01 | 1054 | 39 |
| A200 | ACC | | Jiangmen: Xinhui Gangzhou Hotel | 22.5289, 113.0347 | 2007-09-01 | 1054 | 39 |
| A201 | ACC | | Jiangmen: Xinhui Gangzhou Hotel | 22.5289, 113.0347 | 2007-09-01 | 752 | 39 |
| A202 | ACC | | Jiangmen: Xinhui Gangzhou Hotel | 22.5289, 113.0347 | 2007-09-01 | 1054 | 39 |
| A204 | ACC | | Jiangmen: Xinhui Yujing Hotel | 22.5456, 113.0378 | 2007-09-01 | 752 | 40 |
| A205 | ACC | | Jiangmen: Xinhui Yujing Hotel | 22.5456, 113.0378 | 2007-09-01 | 93 | 41 |
| **Natural isolates (n=59)** | | | | | | | |
| N34 | | Pond | Guangzhou: Huangpu village | 23.0914, 113.3929 | 2006-08-01 | 242 | 42 |
| N36 | | Pond | Guangzhou: Huangpu village | 23.0914, 113.3929 | 2006-08-01 | 739 | 43 |
| N37 | | Pond | Guangzhou: Huangpu village | 23.0914, 113.3929 | 2006-08-01 | 739 | 44 |
| N38 | | Pond | Guangzhou: Huangpu village | 23.0914, 113.3929 | 2006-08-01 | 739 | 45 |
| N39 | | Pond | Guangzhou: Huangpu village | 23.0914, 113.3929 | 2006-08-01 | 739 | 46 |
| N40 | | Pond | Guangzhou: Huangpu village | 23.0914, 113.3929 | 2006-08-01 | 739 | 47 |
| N41 | | Pond | Guangzhou: Huangpu village | 23.0914, 113.3929 | 2006-08-01 | 739 | 48 |
| N43 | | Pond | Guangzhou: Huangpu village | 23.0914, 113.3929 | 2006-08-01 | 739 | 49 |
| N45 | | Pond | Guangzhou: Huangpu village | 23.0914, 113.3929 | 2006-08-01 | 1267 | 50 |
| N47 | | Pond | Guangzhou: Huangpu village | 23.0914, 113.3929 | 2006-08-01 | 1267 | 51 |
| N48 | | Pond | Guangzhou: Huangpu village | 23.0914, 113.3929 | 2006-08-01 | 1267 | 50 |
| N49 | | Pond | Guangzhou: Huangpu village | 23.0914, 113.3929 | 2006-08-01 | 1267 | 50 |
| N50 | | Pond | Guangzhou: Huangpu village | 23.0914, 113.3929 | 2006-08-01 | 1267 | 50 |
| N51 | | Pond | Guangzhou: Huangpu village | 23.0914, 113.3929 | 2006-08-01 | 1267 | 50 |
| N52 | | Pond | Guangzhou: Huangpu village | 23.0914, 113.3929 | 2006-08-01 | 1266 | 52 |
| N53 | | Pond | Guangzhou: Huangpu village | 23.0914, 113.3929 | 2006-08-01 | 1266 | 53 |
| N54 | | Lake | Guangzhou: Liuhua park | 23.1364, 113.2502 | 2006-09-01 | 630 | 54 |
| N56 | | Lake | Guangzhou: Liuhua park | 23.1364, 113.2502 | 2006-09-01 | 630 | 55 |
| N58 | | Lake | Guangzhou: Liuhua park | 23.1364, 113.2502 | 2006-09-01 | 630 | 56 |
| N60 | | Lake | Guangzhou: Liuhua park | 23.1364, 113.2502 | 2006-09-01 | 630 | 57 |
| N62 | | Lake | Guangzhou: Liuhua park | 23.1364, 113.2502 | 2006-09-01 | 1048 | 58 |
| N63 | | Lake | Guangzhou: Liuhua park | 23.1364, 113.2502 | 2006-09-01 | 1782 | 59 |
| N64 | | Lake | Guangzhou: Liuhua park | 23.1364, 113.2502 | 2006-09-01 | 1785 | 60 |
| N65 | | Lake | Guangzhou: Liuhua park | 23.1364, 113.2502 | 2006-09-01 | 1785 | 61 |
| N67 | | Lake | Guangzhou: Liuhua park | 23.1364, 113.2502 | 2006-09-01 | 1788 | 62 |
| N68 | | Lake | Guangzhou: Liuhua park | 23.1364, 113.2502 | 2006-09-01 | 1048 | 63 |
| N69 | | Lake | Guangzhou: Liuhua park | 23.1364, 113.2502 | 2006-09-01 | 1048 | 64 |
| N70 | | Lake | Guangzhou: Liuhua park | 23.1364, 113.2502 | 2006-09-01 | 1048 | 65 |
| N71 | | Lake | Guangzhou: Liuhua park | 23.1364, 113.2502 | 2006-09-01 | 45 | 66 |
| N72 | | Lake | Guangzhou: Liuhua park | 23.1364, 113.2502 | 2006-09-01 | 1048 | 67 |
| N75 | | Lake | Guangzhou: Liuhua park | 23.1364, 113.2502 | 2006-09-01 | 1049 | 68 |
| N83 | | Lake | Guangzhou: Liuhua park | 23.1364, 113.2502 | 2006-09-01 | 752 | 69 |
| N85 | | Lake | Guangzhou: Liuhua park | 23.1364, 113.2502 | 2006-09-01 | 114 | 70 |
| N92 | | Lake | Guangzhou: Liuhua park | 23.1364, 113.2502 | 2006-09-01 | 1051 | 71 |
| N93 | | Lake | Guangzhou: Liuhua park | 23.1364, 113.2502 | 2006-09-01 | 1050 | 72 |
| N95 | | Lake | Guangzhou: Liuhua park | 23.1364, 113.2502 | 2006-09-01 | 1052 | 73 |
| N96 | | Lake | Guangzhou: Liuhua park | 23.1364, 113.2502 | 2006-09-01 | 1053 | 74 |
| N97 | | Lake | Guangzhou: Liuhua park | 23.1364, 113.2502 | 2006-09-01 | 752 | 75 |
| N98 | | Lake | Guangzhou: Liuhua park | 23.1364, 113.2502 | 2006-09-01 | 1048 | 76 |
| N99 | | Lake | Guangzhou: Liuhua park | 23.1364, 113.2502 | 2006-09-01 | 1052 | 77 |
| N102 | | Lake | Guangzhou: Liuhua park | 23.1364, 113.2502 | 2006-09-01 | 1053 | 78 |
| N103 | | Lake | Guangzhou: Liuhua park | 23.1364, 113.2502 | 2006-09-01 | 1053 | 79 |
| N105 | | Lake | Guangzhou: Tianhe park | 23.1276, 113.3676 | 2006-09-01 | 1263 | 20 |
| N108 | | Lake | Guangzhou: Tianhe park | 23.1276, 113.3676 | 2006-09-01 | 1777 | 80 |
| N112 | | Lake | Guangzhou: Tianhe park | 23.1276, 113.3676 | 2006-09-01 | 1049 | 68 |
| N113 | | Lake | Guangzhou: Tianhe park | 23.1276, 113.3676 | 2006-09-01 | 1048 | 81 |
| N114 | | Lake | Guangzhou: Tianhe park | 23.1276, 113.3676 | 2006-09-01 | 1048 | 82 |
| N115 | | Lake | Guangzhou: Tianhe park | 23.1276, 113.3676 | 2006-09-01 | 1048 | 83 |
| N122 | | Lake | Guangzhou: Yuexiu park | 23.1404, 113.2657 | 2006-09-01 | 1777 | 84 |
| N123 | | Lake | Guangzhou: Yuexiu park | 23.1404, 113.2657 | 2006-09-01 | 1048 | 85 |
| N152 | | Lake | Guangzhou: Nanhu | 23.2184, 113.3291 | 2006-11-01 | 1048 | 86 |
| N153 | | Lake | Guangzhou: Nanhu | 23.2184, 113.3291 | 2006-11-01 | 1048 | 82 |
| N166 | | Lake | Guangzhou: Luhu | 23.1472, 113.2821 | 2006-11-01 | 1418 | 87 |
| N207 | | River | Jiangmen: Xinhui Green park | 22.5606, 113.0411 | 2006-11-01 | 1781 | 88 |
| N208 | | River | Jiangmen: Xinhui Green park | 22.5606, 113.0411 | 2006-11-01 | 1 | 89 |
| N209 | | River | Jiangmen: Xinhui Green park | 22.5606, 113.0411 | 2007-10-01 | 1 | 90 |
| N211 | | River | Jiangmen: Xinhui Gangzhou | 22.5292, 113.0347 | 2007-10-01 | 1417 | 35 |
| N212 | | River | Jiangmen: Xinhui Gangzhou | 22.5292, 113.0347 | 2007-10-01 | 1417 | 35 |
| N220 | | Lake | Jiangmen: Xinhui Jade lake | 22.5492, 113.0355 | 2007-10-01 | 45 | 91 |

# ACC indicates air conditioning cooling tower water.

* Geographic location include two cites of Guangdong province (Guangzhou and Jiangmen). Sixteen-six *L .pneumophila* strains were isolated in Guangzhou and 44 were isolated in Jiangmen.
