# Supplementary material for: Molecular typing of Legionella pneumophila isolates from environmental water samples and clinical samples using a five-gene sequence typing and standard Sequence-Based Typing
Source: PLoS One. 2018 Feb 1;13(2):e0190986. doi: 10.1371/journal.pone.0190986 (PMC5794064; doi:10.1371/journal.pone.0190986)
Supplement: S2 Table — (DOCX) [file pone.0190986.s002.docx]

**S2 Table. Sequence variation of the MLST loci and primers for the MLST scheme*.***

| #Gene | $Gene ID | Primers (5’-3’) | Fragment size (bp)  of amplified  product | Size of region used to determine allele type | Gene region used for  allele assignment | No. of allele  types | *No.of variable  sites | % Sequence  variation |
| --- | --- | --- | --- | --- | --- | --- | --- | --- |
| *cca* | 19834308 | ATGAAAGTCTACTTGGTAGGC | 1197 | 1082 | 52-1133 | 17 | 138 | 12.75 |
|  |  | CTCTACACAAGCCACACG |  |  |  |  |  |  |
| *trpA* | 19832870 | ATGAACCGTATTGATAAGACTCTGG | 797 | 748 | 30-777 | 18 | 81 | 10.83 |
|  |  | TCGATAGCTTGCCTCATGGA |  |  |  |  |  |  |
| *lssD* | 19833082 | TCACGGATGAGAGAGTTGAATC | 385 | 330 | 439-768 | 12 | 84 | 25.45 |
|  |  | GCTTCAATTAATAAAGTATCATCAAGCG |  |  |  |  |  |  |
| *lspE* | 19832884 | ACTCTGTCTTCATAAGTTTCAATGTG | 380 | 331 | 66-396 | 17 | 70 | 21.15 |
|  |  | GCCAAAAGCAATGGAATTGTTG |  |  |  |  |  |  |
| *icmK* | 19832016 | GCTGATCAATCAGATGATGCTC | 436 | 385 | 105-489 | 83 | 112 | 29.09 |
|  |  | GTGATAATCTTATTACTGGTGGTG |  |  |  |  |  |  |

* Number of variable sites of the gene fragments were obtained by analyzing 36 to 87 known sequences in NCBI database.

# These gene loci were chosen for MLST scheme.

$ NCBI ID of the target gene of *L. pneumophila* *subsp. pneumophila* str. Philadelphia 1 are shown in this table.
