# Supplementary material for: Molecular typing of Legionella pneumophila isolates from environmental water samples and clinical samples using a five-gene sequence typing and standard Sequence-Based Typing
Source: PLoS One. 2018 Feb 1;13(2):e0190986. doi: 10.1371/journal.pone.0190986 (PMC5794064; doi:10.1371/journal.pone.0190986)
Supplement: S3 Table — (DOCX) [file pone.0190986.s003.docx]

**S3 Table. Number of sequence types and IODs of the environmental isolates achieved by the SBT and the five-gene MLST.**

| Typing methods | No. of types | | | Index of discrimination | | |
| --- | --- | --- | --- | --- | --- | --- |
|  | Artificial isolates | Natural isolates | All isolates | Artificial isolates | Natural isolates | All isolates |
| SBT | 17 | 23 | 33 | 0.807 | 0.914 | 0.920 |
| Five-gene MLST | 41 | 52 | 89 | 0.902 | 0.973 | 0.985 |
