# Supplementary material for: Molecular typing of Legionella pneumophila isolates from environmental water samples and clinical samples using a five-gene sequence typing and standard Sequence-Based Typing
Source: PLoS One. 2018 Feb 1;13(2):e0190986. doi: 10.1371/journal.pone.0190986 (PMC5794064; doi:10.1371/journal.pone.0190986)
Supplement: S4 Table — (DOCX) [file pone.0190986.s004.docx]

**S4 Table. Distribution of environmental *L. pneumophila* isolates in each group of ST or nST.**

| **ST group** | ST | Natural isolates | | Artificial isolates | | *P*-value* |
| --- | --- | --- | --- | --- | --- | --- |
|  |  | n |  | n |  |  |
| Group 1 | 1778,160, 1779, 739, 1049, 1266, 59, 1054, 114, 242, 93 | 15 | N34, N36, N37, N38, N39, N40, N41, N43, N52, N53,N71, N75, N85, N112, N220 | 13 | A3, A4, A5, A7, A25, A26, A189,A195,A196, A197, A200, A202, A205 | 0.994 |
| Group 2 | 1051,1053, 1048, 1050, 1052 | 20 | N62, N68, N69, N70, N72, N92, N93, N95, N96, N98, N99, N102, N103,N113, N114, N115, N123, N152, N153 | 1 | A15 | ***< 0.001*** |
| Group 3 | 1788 | 1 | N67 | 0 | null | 1.000 |
| Group 4 | 1267,1263,1262,1040,1046,630,172,1,752 | 15 | N45, N47, N48, N49, N50, N51, N54, N56, N58, N60,N83, N97, N105, N208, N209 | 33 | A1, A2, A8, A9, A10, A11, A12, A14, A16, A17, A18, A19, A20, A21, A22, A23, A24, A27, A28, A29, A30, A31, A32, A33, A171, A172, A173, A175, A176, A191, A194, A201, A204 | ***< 0.001*** |
| Group 5 | 1417, 1777, 1785, 1418, 1782, 1781 | 9 | N63, N64, N65, N108, N122, N166, N207 N211, N212, | 4 | A6, A180, A181, , A174 | 0.255 |
| **nST group** | nST |  |  |  |  |  |
| Group 1 | 1, 13, 38, 26, 42, 16, 24, 54, 37, 28, 55, 57, 84, 8, 18, 27, 9, 25, 17, 80, 58, 15, 32, 19, 10, 21, 89, 14, 90, 56, 12, 12, 20, 33, 34, 31, 40, 75, 69, 29, 30, 2, 59, 62 | 15 | N34, N54, N56, N60, N122, N108, N62, N208, N209, N58, N105, N97, N83, N63, N67 | 33 | A11, A14, A194, A29, A17, A27, A191, A33, A8, A21, A32, A9, A28, A19, A20, A16, A18, A30, A174, A22, A10, A24, A15, A12, A23, A31, A175, A176, A173, A204, A171, A172, A2 | ***< 0.001*** |
| Group 2 | 43,46, 48, 44, 45, 68, 47, 49, 52, 53, 70, 3, 41, 7, 1, 4, 22, 23, 88, 50, 1, 6, 35, 82, 63, 76, 65, 81, 83, 86, 64, 67, 85, | 32 | N36, N37, N38, N39, N40, N41, N43, N45, N47, N48, N49, N50, N51, N52, N53, N68, N69, N70, N72, N75, N85, N98, N112, N113, N115, N123, N114, N152, N153, N207, N211, N212 | 10 | A1, A3, A4, A7, A25, A26, A6, A180, A181, A205, | ***< 0.001*** |
| Group 3 | 60, 61, 39, 72, 71, 73, 77, 74, 78, 79, 87 | 10 | N64, N65, N92, N93, N95, N96, N99, N102, N103, N166 | 5 | A196, A197, A200, A201, A202 | 0.404 |
| Group 4 | 66, 91, | 2 | N71, N220 | 0 | null | 0.498 |
| Group 5 | 5, 36 | 0 |  | 3 | A5, A189, A195 | 0.096 |

* *P*-value was obtained by Chi-Square test or Fisher’s exact test.
