# Supplementary material for: Molecular typing of Legionella pneumophila isolates from environmental water samples and clinical samples using a five-gene sequence typing and standard Sequence-Based Typing
Source: PLoS One. 2018 Feb 1;13(2):e0190986. doi: 10.1371/journal.pone.0190986 (PMC5794064; doi:10.1371/journal.pone.0190986)
Supplement: S5 Table — (DOCX) [file pone.0190986.s005.docx]

**S5 Table. Environmental ST1 isolates information.**

| SBT sequence type (ST) | Strain source | Strain name | MLST sequence type (nST) |
| --- | --- | --- | --- |
| ST1 | Artificial water | A2 | nST2 |
|  |  | A8 | nST8 |
|  |  | A9 | nST9 |
|  |  | A10 | nST10 |
|  |  | A11 | nST11 |
|  |  | A12 | nST12 |
|  |  | A14 | nST13 |
|  |  | ***A16*** | ***nST17*** |
|  |  | A17 | nST16 |
|  |  | A18 | nST15 |
|  |  | ***A19*** | ***nST17*** |
|  |  | ***A20*** | ***nST17*** |
|  |  | A21 | nST18 |
|  |  | A22 | nST19 |
|  |  | A24 | nST21 |
|  |  | A30 | nST15 |
|  |  | A31 | nST20 |
|  |  | A33 | nST28 |
|  |  | A191 | nST37 |
|  |  | A194 | nST38 |
|  | Natural water | N208 | nST89 |
|  |  | N209 | nST90 |
