# Supplementary figures and images for: Molecular typing of Legionella pneumophila isolates from environmental water samples and clinical samples using a five-gene sequence typing and standard Sequence-Based Typing
Source: PLoS One. 2018 Feb 1;13(2):e0190986. doi: 10.1371/journal.pone.0190986 (PMC5794064; doi:10.1371/journal.pone.0190986)

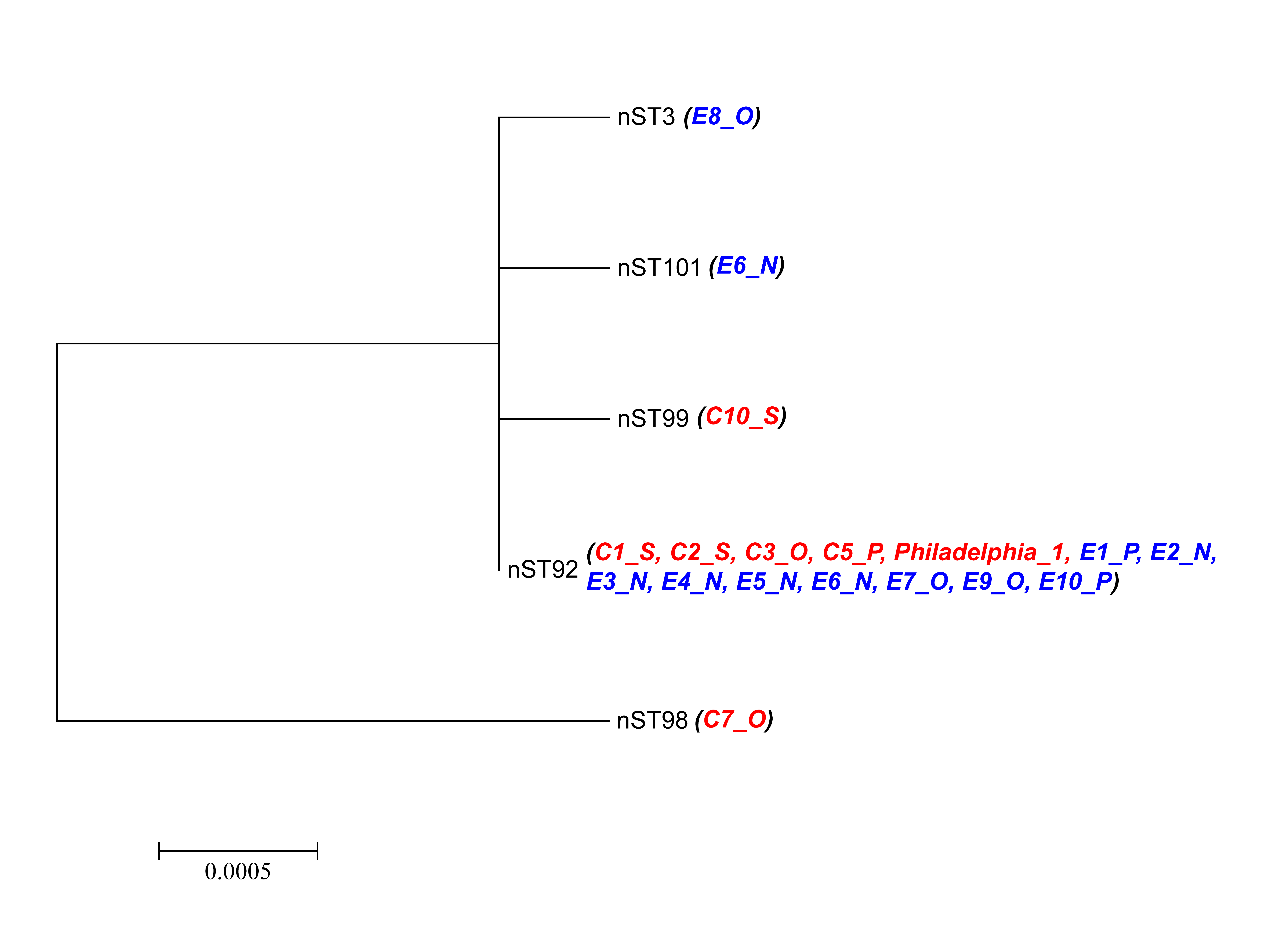

Supplement: S1 Fig — The clinical isolates were marked red, while the environmental isolates were marked blue. (TIF) [file pone.0190986.s006.tif]
